# Targeting Fascin1 maintains chondrocytes phenotype and attenuates osteoarthritis development

**DOI:** 10.1038/s41413-024-00357-1

**Published:** 2024-09-04

**Authors:** Panpan Yang, Yun Xiao, Liangyu Chen, Chengliang Yang, Qinwei Cheng, Honghao Li, Dalin Chen, Junfeng Wu, Zhengquan Liao, Changsheng Yang, Chong Wang, Hong Wang, Bin Huang, Ee Ke, Xiaochun Bai, Kai Li

**Affiliations:** 1 Academy of Orthopedics, Guangdong Province, Guangdong Provincial Key Laboratory of Bone and Joint Degeneration Diseases, The Third Affiliated Hospital of Southern Medical University, Guangzhou, China; 2 Guangxi Key Laboratory of Basic and Translational Research of Bone and Joint Degenerative Diseases, Guangxi Biomedical Materials Engineering Research Center for Bone and Joint Degenerative Diseases, Department of Orthopedics, Affiliated Hospital of Youjiang Medical University for Nationalities, Baise, Guangxi China; 3 School of Mechanical Engineering, Dongguan University of Technology, Dongguan, Guangdong China; 4 Guangdong Provincial People’s Hospital (Guangdong Academy of Medical Sciences), Southern Medical University, Guangdong Lung Cancer Institute, Guangzhou, China; 5 Department of Cell Biology, School of Basic Medical Science, Southern Medical University, Guangzhou, China

**Keywords:** Pathogenesis, Bone

## Abstract

Osteoarthritis (OA) is the most common form of arthritic disease, and phenotypic modification of chondrocytes is an important mechanism that contributes to the loss of cartilage homeostasis. This study identified that Fascin actin-bundling protein 1 (FSCN1) plays a pivotal role in regulating chondrocytes phenotype and maintaining cartilage homeostasis. Proteome-wide screening revealed markedly upregulated FSCN1 protein expression in human OA cartilage. FSCN1 accumulation was confirmed in the superficial layer of OA cartilage from humans and mice, primarily in dedifferentiated-like chondrocytes, associated with enhanced actin stress fiber formation and upregulated type I and III collagens. FSCN1-inducible knockout mice exhibited delayed cartilage degeneration following experimental OA surgery. Mechanistically, FSCN1 promoted actin polymerization and disrupted the inhibition of Decorin on TGF-β1, leading to excessive TGF-β1 production and ALK1/Smad1/5 signaling activation, thus, accelerated chondrocyte dedifferentiation. Intra-articular injection of FSCN1-overexpressing adeno-associated virus exacerbated OA progression in mice, which was mitigated by an ALK1 inhibitor. Moreover, FSCN1 inhibitor NP-G2-044 effectively reduced extracellular matrix degradation in OA mice, cultured human OA chondrocytes, and cartilage explants by suppressing ALK1/Smad1/5 signaling. These findings suggest that targeting FSCN1 represents a promising therapeutic approach for OA.

## Introduction

Osteoarthritis (OA) is the most common disabling arthropathy affecting the entire joint, leading to chronic disability and associated joint dysfunction.^1^ Degradation of articular cartilage is the major characteristic of OA progression, with risk factors including phenotypic changes of chondrocytes, extracellular matrix (ECM) degradation and production of inflammatory cytokines.^2,3^ Chondrocytes, the unique cell type in cartilage, are terminally differentiated cells. Given the complex anisotropic structure and avascularity of articular cartilage, its self-repairing ability in OA is limited.^4^ Chondrocytes with enhanced expression of type I (Col1) and type III (Col3) collagens, accompanied by a significant decrease in expression of aggrecan (Acan) and type II collagen (Col2), have been identified in OA cartilage. These cells are located in the upper middle zone of articular cartilage, and could be related to the so-called dedifferentiated chondrocytes with a shift to a fibroblast-like phenotype.^5,6^

Recent evidence has indicated that OA cartilage destruction is closely related to chondrocyte dedifferentiation. These dedifferentiated chondrocytes produce more catabolic factors (e.g., matrix metalloproteinases [MMPs]) and pro-inflammatory cytokines, leading to cartilage ECM degeneration and OA progression.^7^ Dedifferentiated-like chondrocytes are characterized by various changes, including reduced expression of cartilage ECM components, increased expression of fibroblastic matrix molecules, development of an elongated shape, enhanced actin polymerization, and formation of stress fibers.^8^ Studies have shown that OA chondrocytes exhibit a disorganized actin cytoskeleton and differentially express actin-binding proteins that promote actin polymerization.^9^ Moreover, the development of the dedifferentiated phenotype is mediated by the actin polymerization status.^10,11^ However, the potential role and molecular mechanisms of actin-binding proteins in chondrocytes phenotype shifting and OA pathogenesis remain poorly understood.

Transforming growth factor (TGF)-β plays a crucial role in regulating chondrocyte proliferation, differentiation, and ECM production.^12,13^ TGF-β initiates signal transduction by binding to active type II receptors and activating two different type I receptors, also known as activin receptor-like kinases (ALKs).^14^ The ALK5/Smad2/3 is associated with anabolic events, while the ALK1/Smad1/5 pathway is linked to catabolic events and induces MMPs production. A previous study has noted a shift from ALK5/Smad2/3 to ALK1/Smad1/5 signaling in dedifferentiated-like chondrocytes.^15^ Given the critical role of TGF-β signaling in chondrocyte dedifferentiation and maintenance of cartilage homeostasis, we hypothesize that elucidating the relevant regulatory mechanisms could provide a potential therapeutic strategy for OA.

Fascin actin-bundling protein 1 (FSCN1), an actin-bundling protein that cross-links F-actin microfilaments into tight, parallel bundles, plays critical roles in cell migration, motility, adhesion, and other cellular interactions. It has been identified as an emerging biomarker and potential therapeutic target in various types of human cancers.^16^ In this study, we demonstrated the vital role of FSCN1 in modulating cartilage homeostasis and suggested that targeted inhibition of FSCN1 could serve as a novel potential therapeutic approach for OA.

## Results

### FSCN1 expression is markedly upregulated in OA cartilage, and is mainly in dedifferentiated-like chondrocytes

**Fig. 1:**
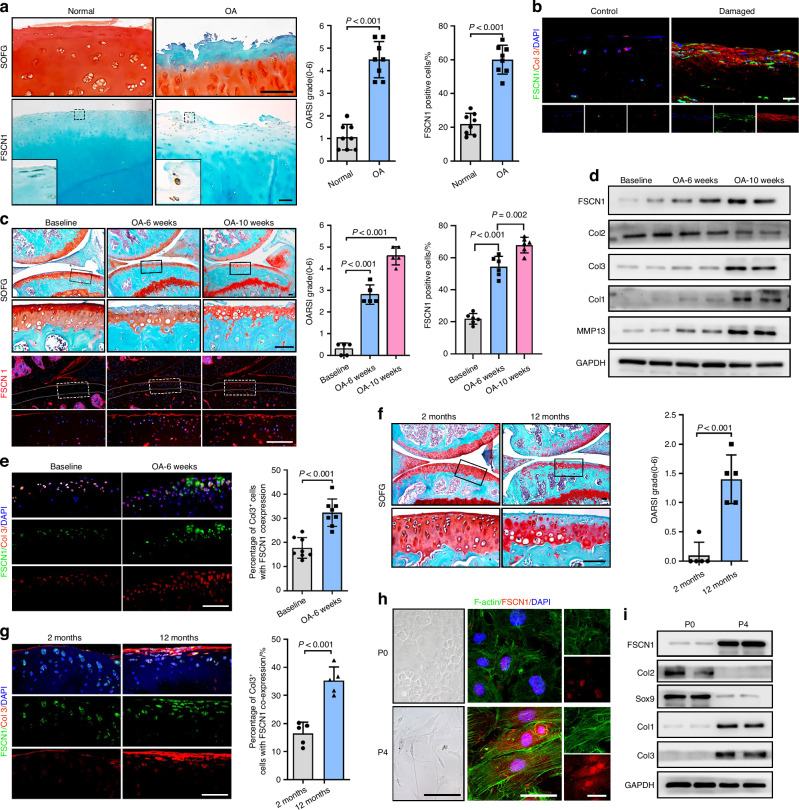
FSCN1 expression is upregulated along with chondrocyte dedifferentiation and OA pathogenesis. **a**, **b** SOFG staining and IHC of FSCN1, Osteoarthritis Research Society International (OARSI) grades and quantification of FSCN1-positive cells (**a**), IF staining of FSCN1 (green) and Collagen type III (red) (**b**) in cartilage from intact (control) and damaged articular cartilage sections collected from OA patients (*n* = 8). **c**, **d** Safranin O/Fast Green (SOFG) staining, immunofluorescence (IF) staining of FSCN1, OARSI grades (**c**), western blot analysis of FSCN1, Collagen type II, MMP13, Collagen type I and III protein levels (**d**) in articular cartilage from mice with baseline or DMM-induced OA at 6- or 10-weeks post-surgery. The inset in each image is shown as a magnified image in the bottom row (*n* = 5). **e** IF staining and quantification of FSCN1 (green) and Collagen type III (red) in articular cartilage from mice with sham or DMM-induced OA at 6 weeks post-surgery (*n* = 5). SOFG staining and OARSI grades (**f**), IF staining of FSCN1 (green) and Collagen type III (red) (**g**) in cartilage from young (2-month-old) and aged (12-month-old) mice (*n* = 5). h-i. Optical microscopy, IF staining of FSCN1 (red) and phalloidin staining of F-actin structures (green) (**h**), western blot analysis of the protein level of FSCN1, Collagen type II, Sox9, Collagen type I and III protein levels (**i**) in mouse primary chondrocytes at passage 0 (P0) and passage 4 (P4) (*n* = 3). Scale bars, 50 μm. All data are presented as means ± SEM

To identify proteins involved in cartilage degeneration, human OA cartilage specimens were collected and analyzed using proteome-wide screening. Among the 2 119 proteins identified, 75 proteins were upregulated and 128 proteins were downregulated (>1.5-fold, *P* < 0.05) in the cartilage of the damaged area compared with the normal area. We observed that FSCN1, an actin-bundling protein, was the most strongly upregulated protein (7.94-fold) in damaged cartilage (Table S1). The marked elevated FSCN1 expression was further confirmed in damaged regions of human articular cartilage compared with undamaged regions by immunostaining. We noted that FSCN1 was predominantly expressed in superficial chondrocytes, which may be dedifferentiated-like chondrocytes (Fig. 1a). Next, we used antibodies against fibroblastic and dedifferentiated-like chondrocytes markers, Col1 and Col3, to co-stain with FSCN1 in human OA cartilage. Results showed that FSCN1 was largely co-localized with Col1 and Col3 in the superficial zone of damaged human OA articular cartilage (Fig. 1b, Fig. S1a). Furthermore, in articular cartilage samples from mice with destabilization of the medial meniscus (DMM) surgery-induced OA, we observed that FSCN1 expression gradually accumulated in chondrocytes as OA progressed, accompanied by diminished expression of type II collagen, and increased levels of MMP13, Col1 and Col3 proteins (Fig. 1c, d). Subsequently, immunofluorescence (IF) staining confirmed that FSCN1 predominantly accumulated in the upper zone of cartilage from experimental OA and aged mice, co-localized with Col1 and Col3 (Fig. 1e-g, Fig. S1b, c). Chondrocyte dedifferentiation is a result of monolayer culture for cell number expansion. Primary chondrocytes from mice were then cultured under different conditions. We observed notable changes in the morphology of chondrocytes over several passages (P), with round-shaped chondrocytes at P0 shifting towards a fibroblast-like form at P4. P4 chondrocytes were elongated and showed an enhanced cytoskeleton with the formation of more actin stress fibers, while the expression of FSCN1 was consistently upregulated (Fig. 1h). We confirmed that the chondrocytes were dedifferentiated at P4 with decreased expression of Col2 and Sox9, while Col1, Col3, and FSCN1, were increased (Fig. 1i). Moreover, the increase of FSCN1 protein level and dedifferentiated phenotype was also observed in mouse chondrocytes stimulated with IL-1β (Fig. S2a, b), subjected to mechanical stress (Fig. S2c, d) and in the human chondrocyte cell line SW1353 with IL-1β stimulation (Fig. S2e). In conclusion, our findings indicate that FSCN1 expression is markedly upregulated in dedifferentiated-like chondrocytes, suggesting a role for the upregulation of FSCN1 in chondrocyte dedifferentiation and OA pathogenesis.

### Targeted deletion of FSCN1 in chondrocytes alleviates OA development in mice

**Fig. 2:**
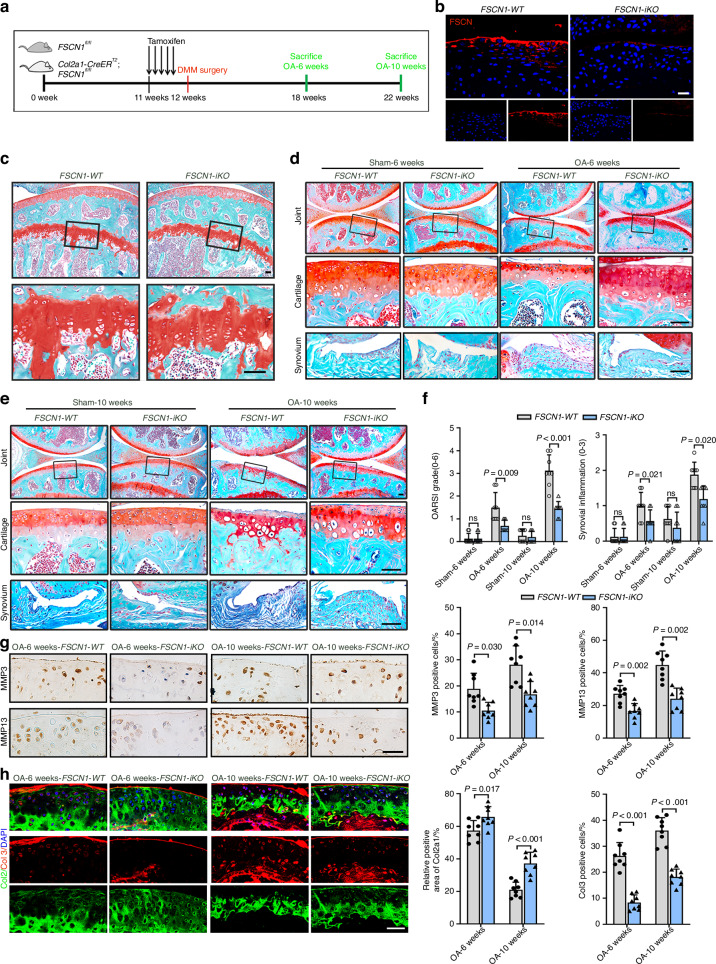
Targeted deletion of FSCN1 in chondrocytes prevents OA development in mice. **a** Twelve-week-old *FSCN1*^*flox*/*flox*^ (*FSCN1*-*WT*) and *FSCN1*-*iKO* mice underwent DMM or sham surgery 1 week after tamoxifen injection. The knees were harvested at 6 or 10 weeks postoperatively for histological analysis (*n* = 8). **b** IF staining of FSCN1 in articular cartilage from *FSCN1*-*WT* and *FSCN1*-*iKO* mice at 6-weeks post-surgery. **c** SOFG staining of joints from *FSCN1*-*WT* and *FSCN1*-*iKO* mice in sham group displayed with growth plate. **d**, **e** SOFG staining of joints from *FSCN1*-*WT* and *FSCN1*-*iKO* mice with sham or DMM-induced OA at 6- or 10-weeks post-surgery. The inset in each image is shown as a magnified image in the bottom row. **f** Cartilage destruction (OARSI grades) and synovial inflammation were determined by SOFG staining and scored (*n* = 8). **g** IHC staining of MMP3 and MMP13 in articular cartilage from *FSCN1*-*WT* and *FSCN1*-*iKO* mice at 6 or 10 weeks post-surgery. **h** IF staining of Collagen type II (green) and III (red) in articular cartilage from *FSCN1*-*WT* and *FSCN1*-*iKO* mice at 6- or 10-weeks post-surgery. Quantified results of each set of data are shown below (*n* = 8). Scale bars, 50 μm. All data are presented as means ± SEM

To determine the effects of FSCN1 on chondrocytes in vivo, we generated mice with conditional deletion of the FSCN1 gene in chondrocytes using *Col2a1-cre* mice. *FSCN1-cKO* homozygous mice exhibited partial neonatal lethality and low survival rate (about 2%). At embryonic day 18.5 (E18.5), *FSCN1-cKO* mice showed smaller skeleton size and delayed mineralization of cranium bone, spine, limb and rib compared to *FSCN1*^*flox*/*flox*^ mice (Fig. S3a). Histologic examination revealed an increased percentage of the proliferation zone and a decreased hypertrophic zone, with significantly reduced mineralization in the limbs of *FSCN1-cKO* mice, as demonstrated by von Kossa staining (Fig. S3b, c). These data suggested that deletion of FSCN1 in chondrocytes led to skeletal abnormalities in mice. To eliminate the developmental defect in *FSCN1-cKO* mice, we then used *Col2a1*-*CreERT2* and *FSCN1*^*flox*/*flox*^ mice to construct inducible conditional knockout mice (*FSCN1*-*iKO*). These mice displayed normal skeletal development and growth plate and were used to examine the role of FSCN1 deletion in DMM surgery-induced OA, as described in the schematic diagram (Fig. 2a–c). As expected, *FSCN1*-*iKO* mice exhibited significantly reduced cartilage destruction, lower OARSI grades, and decreased synovial inflammation compared to *FSCN1*^*flox*/*flox*^ (*FSCN1*-*WT*) mice at both 6 and 10 weeks post-DMM surgery (Fig. 2d–f). Moreover, we observed decreased levels of chondrocyte catabolic markers MMP3 and MMP13, decreased chondrocyte dedifferentiation markers Col3, and elevated chondrocyte anabolism markers Col2 in *FSCN1*-*iKO* mice after DMM surgery (Fig. 2g, h). Thus, our results indicate that targeted deletion of FSCN1 in chondrocytes protects against OA development in vivo.

### FSCN1 promotes chondrocyte dedifferentiation via inducing actin polymerization and activating the ALK1/Smad1/5 signaling

**Fig. 3:**
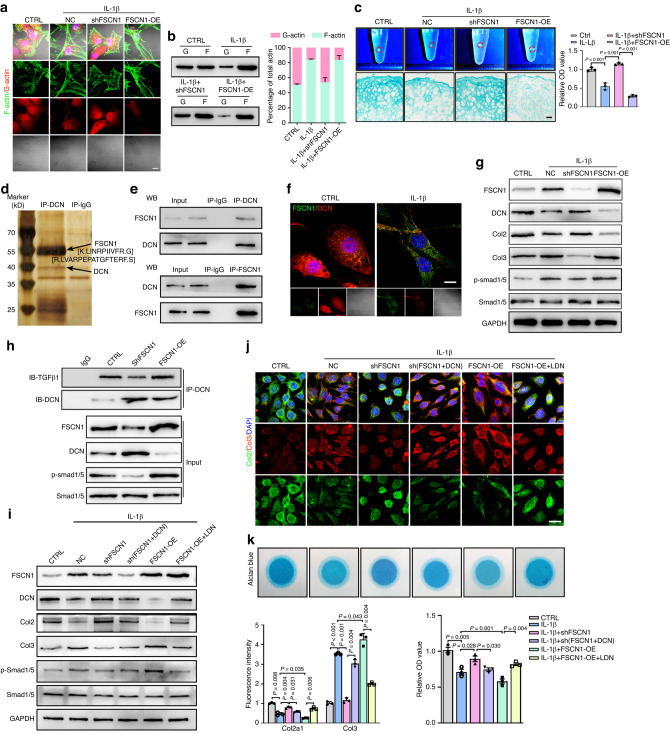
FSCN1 promotes chondrocyte shift towards dedifferentiation phenotype via inducing actin polymerization and activating ALK1/Smad1/5 signaling. **a** Representative images of phalloidin staining of F-actin structures (green) and IF staining for globular (G) actin (red) in chondrocytes with control, IL-1β (10 ng/mL), IL-1β plus FSCN1 knockdown or IL-1β plus FSCN1 overexpression treatment for 24 h. **b** G-/F-actin ratio quantified by western blotting analysis in all groups (*n* = 3). **c** Representative images, alcian blue staining and absorbance quantification of high-density pellet cultured chondrocytes with control, IL-1β (10 ng/mL), IL-1β plus FSCN1 knockdown or IL-1β plus FSCN1 overexpression treatment for 10 days. **d** Cell extracts were subjected to immunoprecipitation with an anti-DCN antibody. The immunoprecipitate–protein complex was separated with SDS–PAGE and the gel was then stained with silver stain. The identified peptide sequences of FSCN1 by LC–MS/MS analysis are shown. **e** FSCN1 or DCN was immunoprecipitated from chondrocytes with an anti-FSCN1 or anti-DCN antibody. The presence of FSCN1 and DCN in these immunoprecipitates was evaluated by immunoblotting. **f** Co-localization of FSCN1 (green) and DCN (red) in chondrocytes with control (saline) or IL-1β (10 ng/mL) treatment for 24 h was analyzed by confocal microscopy. **g** Western blot analysis of FSCN1, DCN, Collagen type II and III, p-Smad1/5 and Smad1/5 in chondrocytes treated with control, IL-1β (10 ng/mL), IL-1β plus FSCN1 knockdown and with IL-1β plus FSCN1 overexpression for 24 h (*n* = 3). **h** Chondrocytes with control, FSCN1 knockdown, or FSCN1 overexpression were subjected to immunoprecipitation with an anti-DCN antibody. The presence of TGF-β1 and DCN in immunoprecipitates was evaluated by immunoblotting. Western blot analysis of FSCN1, DCN, Collagen type II and III, p-Smad1/5 and Smad1/5 (**i**), IF staining and quantification of Collagen type II (green) and III (red) (**j**), alcian blue staining and absorbance quantification (**k**) in chondrocytes with control, IL-1β (10 ng/mL), IL-1β plus FSCN1 knockdown or IL-1β plus FSCN1 and DCN knockdown, IL-1β plus FSCN1 overexpression, IL-1β plus FSCN1 overexpression and LDN-193719 (5 μg/mL) treatment for 48 h (*n* = 3). Scale bars, 50 μm. All data are presented as means  ± SEM

We next investigated the role and underlying mechanism of FSCN1 in chondrocyte dedifferentiation. Proteomic analysis was performed on protein extracted from mouse chondrocytes with or without FSCN1 knockdown by shRNA and treated with IL-1β. Functional enrichment analysis revealed that the biological processes governing actin distribution, including regulation of actin polymerization and depolymerization, were affected (Fig. S4a). Actin plays crucial roles in cell motility through a dynamic process driven by polymerization and depolymerization, that is, the globular to fibrous actin transition.^17^ We observed increased levels of F-actin and decreased G-actin upon IL-1β stimulation. FSCN1 knockdown attenuated this effect, while FSCN1 overexpression exacerbated it, demonstrating that FSCN1 promoted actin polymerization in chondrocytes (Fig. 3a-b). In high-density pellet cultured chondrocytes, FSCN1 knockdown protected against proteoglycan content loss, increased Col2, and decreased Col1 expression compared to the IL-1β treated group. Conversely, FSCN1 overexpression showed opposite changes upon IL-1β stimulation, indicating a shift towards a dedifferentiated phenotype (Fig. 3c, Fig. S4b).

Proteomic analysis identified 261 upregulated and 205 downregulated proteins (>1.5-fold) in FSCN1-knockdown cells, with Decorin (DCN) being the most upregulated protein (12.47-fold) (Table S2, Fig. S4c). Next, we identified FSCN1 as an interacting protein among DCN-immunoprecipitated proteins by proteomic analysis (Fig. 3d), and confirmed the interaction between FSCN1 and DCN in chondrocytes by immunoprecipitation (IP) analyses (Fig. 3e). Double IF staining confirmed the colocalization of FSCN1 and DCN, while IL-1β treatment decreased DCN expression (Fig. 3f). DCN is reported to be a natural inhibitor of TGF-β and an essential constituent of the native cartilage matrix.^18^ Next, we noticed that IL-1β treatment could increase Col3 expression and Smad1/5 phosphorylation, accompanied by inhibited DCN, Col2 expression, and increased TGF-β1 in the supernatant. Moreover, FSCN1 knockdown attenuated these effects, while FSCN1 overexpression exacerbated them (Fig. 3g, Fig. S4d). IP analyses showed that DCN binding to TGF-β1 was enhanced by FSCN1 deletion but diminished by FSCN1 overexpression (Fig. 3h). The effects of FSCN1 knockdown on Col2, Col3, phosphorylated Smad1/5, and proteoglycan content were reversed by DCN knockdown. Conversely, ALK1 inhibitor LDN-193719 mitigated the effects of FSCN1 overexpression upon IL-1β stimulation (Fig. 3i–k). In *FSCN1-cKO* mice, DCN, Col2 and Sox9 expression were upregulated, while p-Smad1/5 expression was suppressed compared to *FSCN1*^*floxp*/*flox*^ mice (Fig. S5a, b). *FSCN1*-*iKO* mice showed upregulated DCN expression and downregulated nuclear expression of p-Smad1/5 and β-catenin in joint sections at both 6- and 10-weeks post-DMM surgery (Fig. S6a, b). Taken together, these data demonstrate that FSCN1 promotes actin polymerization and interrupts DCN inhibition of TGF-β1, activating ALK1/Smad1/5 signaling, thus expediting chondrocytes shift towards a dedifferentiation phenotype.

### Local injection of FSCN1-overexpressing adeno-associated virus exacerbates OA progression through activation of the ALK1/Smad1/5 signaling in vivo

**Fig. 4:**
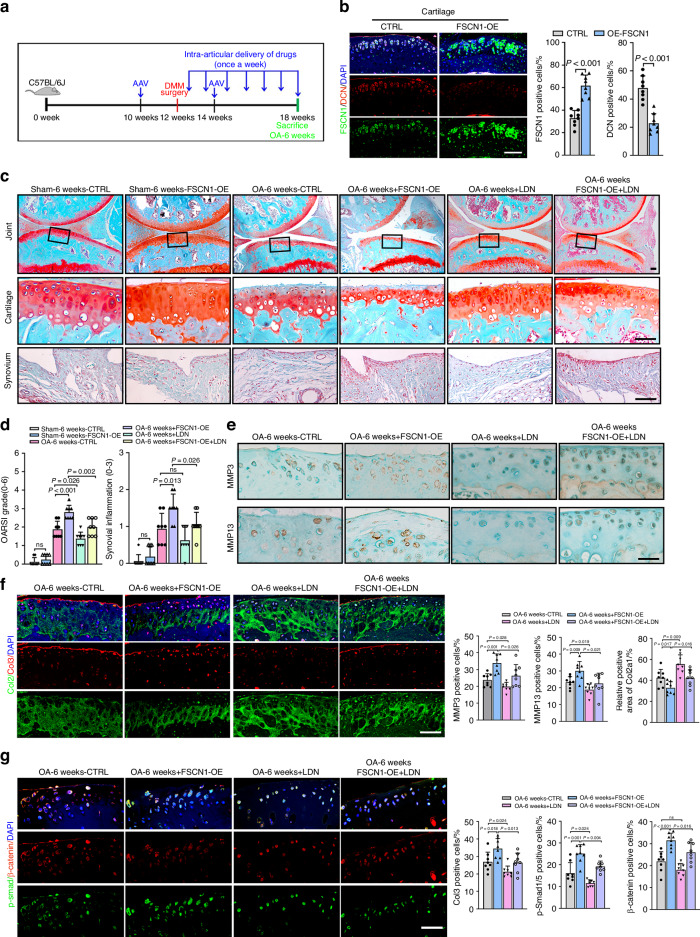
Local injection of FSCN1 overexpressing adeno-associated virus exacerbates OA progression through activation of the ALK1/Smad1/5 signaling in vivo. **a** Twelve-week-old C57BL6/J mice underwent DMM or sham surgery, AAV-FSCN1 or AAV-control was injected into the knee joints at ten and fourteen week age, and saline (control), or LDN-193719 (20 mmol/L) was injected into the knee joints every week after surgery. The knees were harvested at 6 weeks postoperatively for histological analysis (*n* = 8). **b** IF staining and quantification of FSCN1 (red) and DCN (green) in articular cartilage from control and FSCN1-OE mice at 6 weeks post-surgery (*n* = 8). **c** SOFG staining of joints from mice with DMM-induced OA in control, FSCN1-OE, LDN-193719 and FSCN1-OE with LDN-193719 group at 6 weeks post-surgery. The insets in the images are shown as magnified images in the bottom row (*n* = 8). **d** Cartilage destruction (OARSI grades) and synovial inflammation were determined by SOFG staining and scored (*n* = 8). **e** IHC staining of MMP3 and MMP13 in articular cartilage from mice with DMM-induced OA in all groups at 6 weeks post-surgery. **f**,**g** IF staining of Collagen type II (green) and III (red) (**f**), p-Smad1/5 (green) and β-catenin (red) (**g**) in articular cartilage from mice with DMM-induced OA in all groups at 6 weeks post-surgery. Quantified results of each data set are shown below (*n* = 8). Scale bars, 50 μm. All data are presented as means ± SEM

Next, we sought to examine the effect of FSCN1 overexpression on OA progression via intra-articular injection of FSCN1-overexpressing adeno-associated virus (AAV) and to determine whether this effect could be rescued by ALK1/Smad1/5 signaling inhibitor. Mice were treated as shown in the diagram (Fig. 4a). IF staining confirmed FSCN1 overexpression in articular cartilage, while DCN expression was inhibited in mice receiving AAV injection (Fig. 4b). Intra-articular injection of control or FSCN1-overexpressing AAV did not affect the normal joint structure in the sham group. Histological examination revealed that cartilage from control mice began to lose proteoglycan and develop fibrillation at the cartilage surface, with synovitis emerging at 6 weeks post-surgery. FSCN1-overexpressing (FSCN1-OE) mice exhibited greater loss of proteoglycans, more severe cartilage erosion beyond the tidemark, roughness of the cartilage surface or clefts, and increased synovitis after DMM surgery, as confirmed by elevated OARSI and synovitis scores. Moreover, treatment with ALK1 inhibitor LDN-193719 attenuated both cartilage destruction and synovitis inflammation caused by FSCN1-OE (Fig. 4c, d). Accordingly, expression of chondrocyte catabolic markers (MMP3, MMP13) and dedifferentiation marker (Col3) were elevated, while the anabolic marker (Col2) was decreased in FSCN1-OE mice after DMM surgery. The ALK1 inhibitor-treated group showed an improved trend (Fig. 4e, f). Furthermore, enhanced expression of p-Smad1/5 and β-catenin in chondrocytes were observed in FSCN1-OE mice and was diminished with ALK1 inhibitor treatment (Fig. 4g). Cumulatively, these data confirm that overexpression of FSCN1 exacerbated OA progression, and can be mitigated by ALK1 inhibition, suggesting that FSCN1 activates ALK1/Smad1/5 signaling in vivo.

### FSCN1 inhibitor NP-G2-044 protects chondrocytes against dedifferentiation in vitro via suppressing ALK1/Smad1/5 signaling

**Fig. 5:**
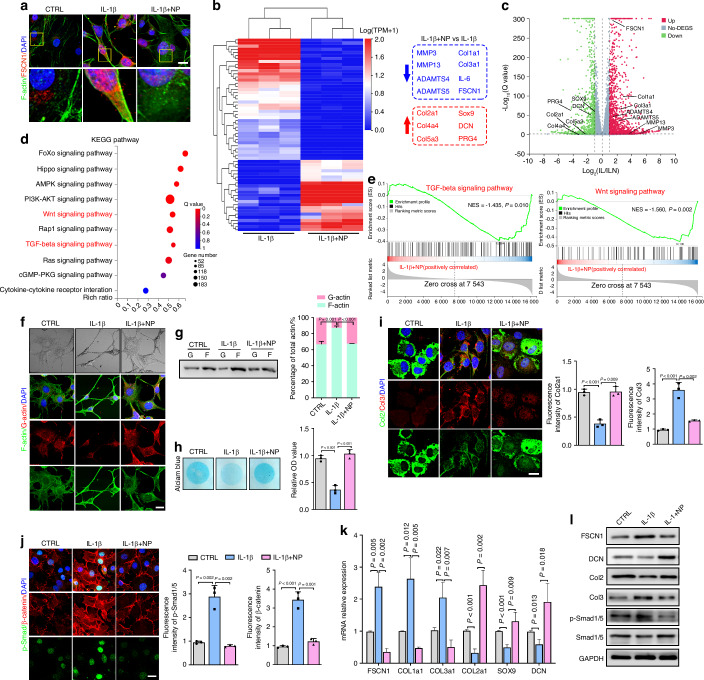
FSCN1 inhibitor NP-G2-044 protects chondrocytes against dedifferentiation in vitro via suppressing ALK1/Smad1/5 signaling. **a** IF staining of FSCN1 (red) and phalloidin (green) staining for F-actin structures in chondrocytes treated with control, IL-1β (10 ng/mL), or IL-1β plus NP-G2-044 (10 μmol/L) for 24 h. Heat map (**b**) and volcano plot (**c**) showing differentially-expressed genes (DEGs) (fold change > 2 or <0.5) from RNA-sequencing of chondrocytes treated with IL-1β or IL-1β plus NP-G2-044; *n* = 3 per condition and seven independent experiments were analyzed. KEGG pathway (**d**) and GSEA (**e**) analysis for DRGs demonstrating TGF-β and Wnt signaling pathway enrichment in chondrocytes treated with IL-1β or IL-1β plus NP-G2-044. **f** Images of optical microscopy, phalloidin staining for F-actin structures (green) and IF staining for globular actin (red) in chondrocytes treated with control, IL-1β (10 ng/mL), or IL-1β plus NP-G2-044 (10 μmol/L) for 24 h. **g** G-/F-actin ratio quantified by western blotting analysis; *n* = 3 independent experiments. Alcian blue staining and absorbance quantification (**h**), IF staining and quantification of Collagen II (green) and III (red) (**i**), IF staining and quantification of p-Smad1/5 (green) and β-catenin (red) (**j**) in chondrocytes treated with control, IL-1β, or IL-1β plus NP for 24 h (*n* = 3). Relative mRNA expression of chondrogenic and dedifferentiation genes (**k**), Western blot analysis of FSCN1, DCN, Collagen type II and III, p-Smad1/5 and Smad1/5 (**l**) in chondrocytes treated with control, IL-1β, or IL-1β plus NP-G2-044 for 24 h (*n* = 3). Scale bars, 50 μm. All data are presented as means ± SEM

Subsequently, we identified a FSCN1 inhibitor, NP-G2-044, currently in phase Ib/IIa clinical trials for treating tumor invasion or metastasis.^19^ Next, mouse chondrocytes were treated with IL-1β with or without NP-G2-044. We verified that IL-1β enhanced the interaction between FSCN1 and F-actin, while NP-G2-044 inhibited this process (Fig. 5a). RNA-sequencing analysis was performed on chondrocytes treated with IL-1β or IL-1β plus NP-G2-044 stimulation. Lists of all significantly upregulated genes (URGs) and downregulated genes (DRGs) in each comparison are provided in supplementary Table S3, where genes with a false discovery rate of <0.05 and a |log_2_(fold change (FC)) |>1 were considered to be significantly differentially-expressed genes. NP-G2-044 treatment significantly upregulated cartilage ECM genes (Col2a1, Sox9, Col4a1, Col5a3, DCN, PRG4) and downregulated OA-associated genes (MMP3, MMP13, ADAMTS4/5). Notably, chondrocyte dedifferentiation-related genes (Col1a1, Col3a1, IL-6, FSCN1) were all significantly decreased with NP-G2-044 treatment (Fig. 5b, c). Furthermore, Kyoto encyclopedia of genes and genomes (KEGG) and gene set enrichment analysis (GSEA) analyses revealed that TGF-β, Wnt, cellular senescence, collagen fibril organization signaling and rheumatoid arthritis pathways were markedly downregulated in IL-1β plus NP-G2-044-stimulated chondrocytes (Fig. 5d, e, Fig. S7a, b). These data indicated that NP-G2-044 showed potential protective effect against chondrocytes dedifferentiation in vitro. Further, NP-G2-044 treatment inhibited the transition of G-actin to F-actin and the flattened cell shape caused by IL-1β stimulation, maintained a more rounded morphology (Fig. 5f, g). Alcian blue staining showed that NP-G2-044 treatment preserved proteoglycan content despite IL-1β stimulation (Fig. 5h). IF staining revealed that inhibition of FSCN1 with NP-G2-044 markedly increased Col2 and decreased Col3 expression, while reducing nuclear expression of p-Smad1/5 and β-catenin compared to the IL-1β-treated group (Fig. 5i, j). RT-qPCR analysis confirmed the up-regulation of chondrogenic genes and down-regulation of dedifferentiation genes following NP-G2-044 treatment (Fig. 5k). Moreover, immunoblotting analysis confirmed that NP-G2-044 rescued the chondrocytes from dedifferentiation after IL-1β stimulation, increased Col2 and DCN expression, decreased Col3 expression, and inhibited ALK1/Smad1/5 signaling (Fig. 5l). All together, we confirm that inhibition of FSCN1 with NP-G2-044 protects against chondrocytes dedifferentiation by suppressing ALK1/Smad1/5 signaling in vitro, suggesting NP-G2-044 may have therapeutic potential for OA.

### FSCN1 inhibitor NP-G2-044 ameliorates OA progression at the onset, early and middle stages in mice

**Fig. 6:**
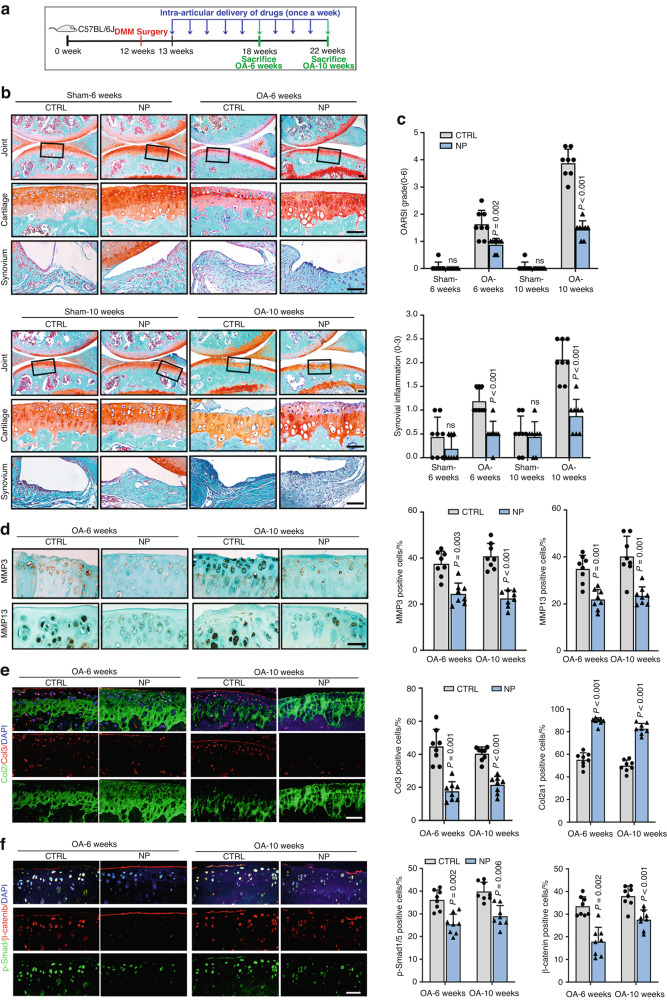
FSCN1 inhibitor NP-G2-044 ameliorates OA progression at early and middle stages in mice. **a** Twelve-week-old C57BL6/J mice underwent DMM or sham surgery, and saline (control), or NP-G2-044 (10 mmol/L) was injected into the knee joints every week after surgery. The knees were harvested at 6 or 10 weeks postoperatively for histological analysis (*n* = 8). **b** SOFG staining of joints from mice with DMM-induced OA and treated with control or with NP-G2-044 at 6 or 10 weeks post-surgery. The insets in the images are shown as magnified images in the bottom row. **c** cartilage destruction (OARSI grades) and synovial inflammation were determined by SOFG staining and scored (*n* = 8). IHC staining of MMP3 and MMP13 (**d**), IF staining of Collagen type II (green) and III (red) (**e**) and p-Smad1/5 (green) and β-catenin (red) (**f**) in articular cartilage from control mice and those treated with NP-G2-044 at 6 or 10 weeks post-surgery (*n* = 8). Scale bars, 50 μm. All data are presented as means ± SEM

To directly assess the therapeutic effects of FSCN1 inhibitor against OA, mice underwent DMM surgery followed by intra-articular injection of NP-G2-044 or phosphate-buffered saline (PBS) as a control. Knee joints were harvested at 4, 6 or 10 weeks post-surgery, as illustrated in Fig. 6a and Supplementary Fig. S8a. Firstly, at 4 weeks after surgery, the NP-G2-044 treatment group displayed preventive effects on cartilage degeneration compared to the control group, indicating a protective role of NP-G2-044 on the onset of OA in mice (Fig. S8b, c). Cartilage from NP-G2-044 treated mice showed diminished expression of FSCN1, chondrocyte catabolic markers (MMP3, MMP13), dedifferentiation marker (Col3), p-Smad1/5 and β-catenin, along with enhanced expression of DCN and chondrocyte anabolism markers (Col2) (Fig. S8d–g). Moreover, for the early and middle-stage OA at 6 and 10 weeks post-surgery in mice, the NP-G2-044 treatment group also displayed desired preventive effects on cartilage degeneration and even synovial inflammation compared to the control group, as reflected by OARSI and synovial inflammation scores (Fig. 6b, c). IF staining confirmed decreased FSCN1 expression and elevated DCN expression in cartilage from NP-G2-044 treated mice (Fig. S9a). Furthermore, the expression of chondrocyte catabolic markers (MMP3, MMP13), dedifferentiation markers (Col3, Col1), p-Smad1/5 and β-catenin significantly decreased, while expression of chondrocyte anabolism markers (Col2a1, Acan) increased in NP-G2-044 treated mice (Fig. 6d–f, Fig. S9b). Thus, we conclude that FSCN1 inhibitor NP-G2-044 can alleviate the progression of experimental OA in mice at the onset, early and middle stages.

### FSCN1 serves as a potential therapeutic target for human OA

**Fig. 7:**
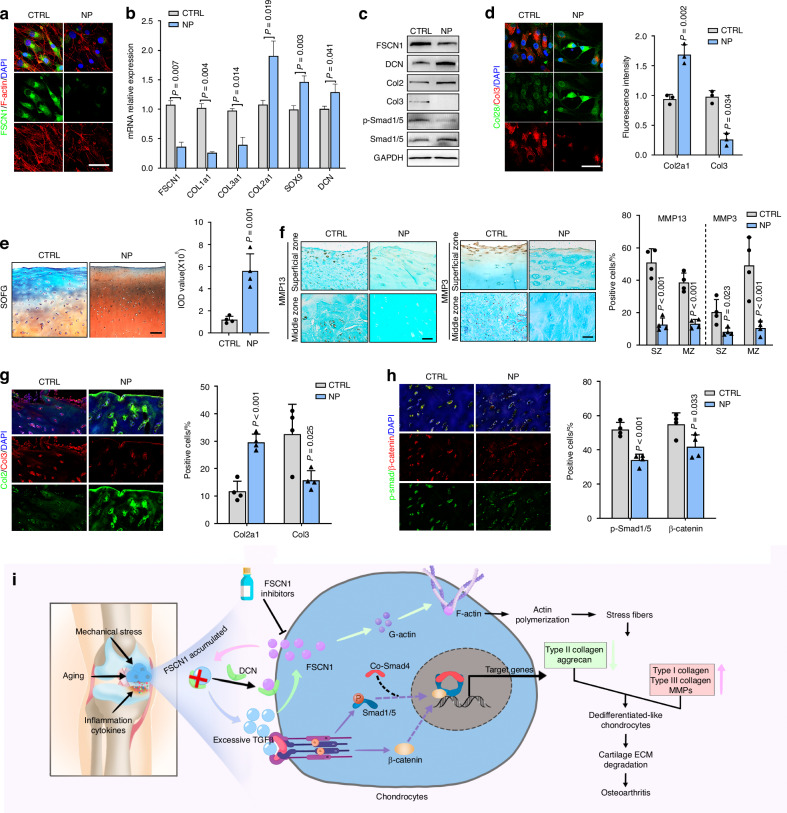
FSCN1 serves as a potential therapeutic target for human OA. IF staining of FSCN1 (green) and phalloidin (red) staining for F-actin structures (**a**), relative mRNA expression of chondrogenic and dedifferentiation genes (**b**), western blot analysis of FSCN1, DCN, Collagen type II and III, p-Smad1/5 and Smad1/5 (**c**), IF staining and quantification of Collagen II (green) and III (red) (**d**) in human chondrocytes treated with control or NP-G2-044 (10 μmol/L) for 24 h (*n* = 3). **e** SOFG staining and absorbance quantification in explant-cultured cartilage tissue from OA donors treated with control or NP-G2-044 (10 μmol/L) for 7 days (*n* = 4). f-h. IHC staining and quantification of MMP13 and MMP3 (**f**), IF staining and quantification of Collagen type II (green) and III (red) (**g**), p-Smad1/5 (green) and β-catenin (red) (**h**) in explant-cultured cartilage tissue from OA doners treated with control or NP-G2-044 (10 μmol/L) for 7 days (*n* = 4). Scale bars, 100 μm. All data are presented as means ± SEM. **i** Schematic diagram showing the role and mechanism of FSCN1 in the regulation of chondrocyte dedifferentiation and the pathogenesis of OA

We next evaluated the potential clinical therapeutic efficacy of NP-G2-044 using primary cultured human articular chondrocytes and tissue explants from patients undergoing total knee arthroplasty. We noticed that, compared with the control group, IF staining revealed inhibition of FSCN1 and decreased binding to F-actin with NP-G2-044 (Fig. 7a). RT-qPCR and immunoblotting analysis displayed that NP-G2-044 rescued the human OA chondrocytes with increased Col2 and DCN expression, decreased Col3 expression and inhibited downstream factors of ALK1/Smad1/5 signaling (Fig. 7b–c). IF staining confirmed enhanced Col2 and decreased Col3 expression with NP-G2-044 treatment (Fig. 7d). Next, cartilage explants were cultured with saline (control) or NP-G2-044 for 7 days. Safranin O and Fast Green staining revealed enhanced preservation of proteoglycan content with NP-G2-044 treatment (Fig. 7e). IHC and IF staining showed decreased MMP13 and MMP3 expression, increased expression of the chondrocyte anabolic marker (Col2), and decreased dedifferentiation marker (Col3) in NP-G2-044-treated samples (Fig. 7f, g). Additionally, inhibited expression of p-Smad1/5 and β-catenin were also observed (Fig. 7h). Overall, the FSCN1 inhibitor NP-G2-044 protects against ECM degradation in human OA tissues (in vitro) and shows potential clinical therapeutic efficacy.

## Discussion

Cartilage destruction and abnormal homeostasis are hallmarks of OA, and halting the progressive degeneration of cartilage is considered an effective therapeutic target.^20^ As the sole cell type in cartilage, chondrocytes play a crucial role in maintaining cartilage homeostasis. Increasing evidence suggests that phenotypic modification of articular chondrocytes contributes to the loss of cartilage homeostasis and may be an early event in the onset of OA.^21^ Studies have indicated that chondrocytes acquire a dedifferentiated phenotype in the upper zone of articular cartilage. This observation is strongly related to the fact that cartilage degenerative changes begin with cellular disturbances at the superficial layer, potentially leading to fibrosis or aging in OA cartilage. These changes continue to hinder the application of cartilage regenerative therapy.^7^ Therefore, understanding the detailed mechanisms of chondrocyte dedifferentiation and maintaining the differentiated phenotype is essential for developing effective OA therapies. In the present study, we demonstrated that FSCN1 accelerates chondrocyte dedifferentiation and cartilage damage by promoting actin polymerization and interrupting the inhibition of DCN on TGF-β1. This leads to excessive TGF-β1 and activation of ALK1/Smad1/5 signaling. Our findings establish the vital role of FSCN1 in chondrocytes phenotype shifting, ECM degradation, and OA progression (Fig. 7i).

Dedifferentiation is a process in which well-differentiated mature cells gradually lose their differentiated phenotype and transform into undifferentiated cells.^22^ Several factors known to drive OA progression, such as pro-inflammatory cytokines (IL-1β or TNF-α), oxidative stress, mechanical stress, and aging, can induce the chondrocyte phenotype switching to a fibroblast-like dedifferentiated state, both in vivo and in vitro.^23^ Dedifferentiated chondrocytes cease expressing Acan and Col2, instead shifting to express Col1 and Col3. They are characterized by an elongated shape, increased actin polymerization, development of stress fibers, and expression of contractile molecules. Consequently, the assembly, integrity, and stability of the ECM are compromised, leading to cartilage fibrosis and OA progression. Previous studies have reported that dedifferentiated chondrocytes in monolayer culture show remarkable changes in cytoskeletal organization, developing well-defined stress fibers resulting in an elongated phenotype.^8^ In our cultures of primary mouse chondrocytes, we observed that chondrocytes displayed a fibroblast-like elongated phenotype with increased passage times during monolayer culture.^10^ We next induced chondrocyte dedifferentiation through passaging, IL-1β treatment, and mechanical stress stimulation, as previously reported. We found that F-actin was enhanced with more stress fibers, while the actin-bundling protein FSCN1 was correspondingly elevated in vitro. In vivo, we tracked dedifferentiated-like chondrocytes by staining for collagen type I and III in cartilage from OA mice and patients. We observed that the number of these cells positively correlated with the degree of OA, as reported in the literature. Notably, these cells were the primary sites of FSCN1 staining. These results prompted us to explore the role and underlying mechanism of FSCN1 in chondrocyte dedifferentiation and OA progression.

The architecture of the actin cytoskeleton is a major phenotype modulator of chondrocytes during dedifferentiation.^24^ Actin depolymerization agents, such as cytochalasin D or latrunculin B, can prevent the dedifferentiation process and promote the re‐expression of chondrogenic genes.^25,26^ Previous studies have demonstrated that the interdependence of cytoskeletal organization and chondrogenic gene expression is regulated by actin-binding proteins, such as adseverin.^27,28^ In our study, we found that deletion of FSCN1 or treatment with FSCN1 inhibitor reversed the upregulated G-/F-actin ratio induced by IL-1β stimulation. These findings indicate that FSCN1 promotes actin polymerization and dedifferentiation in chondrocytes. However, mice with conditional deletion of the FSCN1 gene in chondrocytes displayed skeletal abnormalities and disorganized growth plate compared to control mice. This observation suggests that FSCN1 may have different roles in articular and growth plate chondrocytes.

Among the proteins identified by proteomic analysis of FSCN1-knockdown chondrocytes, the most upregulated expression of DCN caught our attention. DCN is one of the natural inhibitors of TGF-β1, a small leucine-rich proteoglycan that binds with high affinity to this cytokine and prevents its interaction with pro-fibrotic receptors.^29,30^ Recent evidence has shown that DCN plays a protective role in liver fibrogenesis. Its genetic ablation in mice leads to enhanced matrix deposition, impaired matrix degradation, and “activation” of hepatic stellate cells, the main producers of fibrotic tissue.^31^ Moreover, in mice with post-traumatic OA, the absence of DCN resulted in accelerated sulfated glycosaminoglycan loss, formation of highly aligned collagen fibrils on the cartilage surface, and more severe OA.^32,33^ While the direct relationship between FSCN1 and DCN is currently not well understood, various studies suggest that FSCN1 may affect the degradation of the ECM component DCN by regulating the expression of MMPs, influencing cell cytoskeleton remodeling, and other factors.^34–36^ The specific interaction between these proteins requires further investigation.

TGF-β signaling is a key driver of fibrosis in the kidney and other organs,^37^ and plays fundamental roles in chondrocyte biology and OA development.^38^ β-catenin has been reported to accumulate during dedifferentiation in rabbit and human chondrocytes. Ectopic expression of β-catenin or inhibition of its degradation with proteasome inhibitors has been shown to cause dedifferentiation of chondrocytes.^36,39^ Additionally, TGF-β also increases nuclear accumulation and stability of β-catenin.^40^ The ALK5/Smad2/3 pathway is critical in maintaining articular cartilage homeostasis, while a strong correlation between ALK1/Smad1/5 and catabolic events inducing MMPs production has been demonstrated.^41^ Blaney Davidson et al. discovered a change in the TGF-β signaling pathway in cartilage from aging mice, characterized by an increased ALK1/ALK5 ratio. This shift led to enhanced MMP-13 expression and collagen degradation, hallmarks of an OA-like phenotype.^42^ Moreover, during chondrocyte dedifferentiation, the ALK1/ALK5 balance was also shifted, with decreased expression of ALK5 and p-Smad2/3 accompanied by increased ALK1 and p-Smad1/5 expression.^15^ In our study, we confirmed that FSCN1 inhibited the binding of DCN with TGFβ1, and that FSCN1 deletion increased DCN expression while decreasing p-Smad1/5 expression. Thus, these findings suggest that increased FSCN1 protein may downregulate DCN expression, thereby facilitating excessive TGF-β1 activation of ALK1/Smad1/5 signaling. This observation aligns with a previous report.^43^

Currently, no curative treatments are clinically approved for OA, highlighting the urgent need for a detailed understanding of disease pathogenesis and identification of new drug targets.^24^ Our study demonstrates that both conditional knockout of FSCN1 in chondrocytes and treatment with FSCN1 inhibitors conferred chondroprotective effects and protect cartilage from matrix degradation in a DMM-induced OA murine model, suggesting that FSCN1 could be a potential drug target for OA treatment. Furthermore, in cultured cartilage explants from OA patients, FSCN1 inhibitor also slowed down the degradation of proteoglycan contents. The inhibitor used in this study, NP-G2-044, is currently under phase Ib/IIa clinical study (Clinicaltrials.gov NCT05023486) and has shown promise in alleviating OA symptoms to a certain extent. This presents an attractive potential for treating OA. The pharmacokinetic characteristics of NP-G2-044 have been demonstrated promising results in both preclinical and clinical studies, exhibiting an exceptional safety profile with no instances of dose-limiting toxicities, drug-related serious adverse events, or patient fatalities observed. Previous studies have reported that FSCN1 contributes to rheumatoid arthritis development, and inhibition of FSCN1 could suppress the migration and invasion of fibroblast-like synoviocytes.^44,45^ Consistent with these findings, we also observed improvement in synovial inflammation in vivo treatment with FSCN1 inhibitor NP-G2-044. Thus, our data suggest that pharmaceutical targeting of FSCN1 inhibition can be a promising clinical therapeutic approach for OA.

One limitation of this study is that we did not explore the effect and mechanism of targeting FSCN1 in other joint tissues beyond cartilage. This aspect warrants further investigation to provide a more comprehensive understanding of FSCN1’s role in OA pathogenesis. Secondly, our research did not analyze in detail the effects of different doses of FSCN1 inhibitors on OA progression. Future studies should focus on identifying the optimal dosage, administration route, and duration of treatment to achieve the best therapeutic effects against ECM degradation and OA progression. This dose-response analysis will be crucial for translating our findings into clinical applications. Lastly, while our results in murine models are promising, studies in larger animal models and sequential phase clinical trials are necessary to demonstrate the ultimate safety and efficacy of FSCN1 inhibitors in human OA. These studies will help to address potential species-specific differences and provide more robust evidence for the clinical potential of FSCN1 inhibition in OA treatment.

In summary, our study demonstrates that FSCN1 is a key regulator controlling chondrocytes phenotype shifting and ECM degradation by activating ALK1/Smad1/5 signaling. Deletion of FSCN1 in chondrocytes and pharmacological inhibition of FSCN1 effectively ameliorate cartilage degradation in OA mice, validating the potential therapeutic target on OA progression. Importantly, the FSCN1 inhibitor NP-G2-044 showed promising ECM preservation outcomes in ex vivo cultured human OA cartilage, highlighting its translational potential for clinical use. Collectively, our findings provide novel insights for the development of future OA therapies aimed to maintain chondrocytes phenotype and cartilage homeostasis, and FSCN1 is a promising drug target for OA treatment.

## Materials and methods

### Mice and experimental OA

All animal experiments were approved by The Third Affiliated Hospital of Southern Medical University and the Academy of Orthopedics in Guangdong province. All *C57BL/6J* and transgenic mice were housed in a specific-pathogen-free level animal room in an environment with a temperature of 20 °C–26 °C and a humidity of 50%–60%, and a 12-h light/dark cycle was maintained. We used *Cre* recombinase-mediated *Fscn1* gene recombination to construct chondrocyte-specific *Fscn1* knockout transgenic mice. *Fscn1*-flox (Strain #:T019490, purchased from GemPharmatech, Nanjing, China) homozygous females and *Col2*-CreER (Strain #:NM-KI-18029, purchased from Shanghai Model Organisms Center, Inc., Shanghai, China) or *Col2*-*Cre* transgenic males (Strain #:003554, purchased from Jackson Laboratories, Bar Harbor, ME, USA) were crossed to generate *Col2*-*Cre*: *Fscn1*^flox/flox^ mice (*FSCN1-cKO*) or *Col2*-*CreER*: *Fscn1*^flox/flox^ mice (*FSCN1*-*iKO*); *Cre*-negative *Fscn1*^flox/flox^ mice (termed *Fscn1*^flox/flox^) served as controls. Tamoxifen (0.1 mg/g body weight) was administered to the 8-week-old mice via intraperitoneal injection (once per day for 5 consecutive days) to induce the *Cre*-recombinase-mediated deletion of *Fscn1* in Col2-expressing cells. DNA isolated from mouse tails was used to perform PCR to identify the genotypes of transgenic mice with the primers for *Col2*-*Cre*, *Col2*-*CreER*, and *Fscn1* *loxp* (Table S5). Mice were sacrificed at embryonic day 18.5 for histological analysis of the knee joint. *Fscn1*^flox/flox^ mice (*Cre*-negative control) were injected with tamoxifen in the same way. All transgenic mice had been backcrossed on the C57BL/6JGpt strain (Strain #: N000013, purchased from GemPharmatech, Nanjing, China) for at least ten generations. We only used male mice because tamoxifen had been widely thought to have effects in female mice, including on cartilage, and male mice were more prone to OA. Post-traumatic OA was induced by destabilization of the medial meniscus (DMM) surgery in 12-week-old male mice.^46^ Sham-operated mice of the same sex and age were used as controls. Briefly, the meniscus ligament was located and cut in the DMM surgery group. However, in the sham group, no resection was performed after finding the meniscal tibial ligament. Mice were sacrificed by overdose anesthesia at 4, 6 or 10 weeks after DMM surgery. Knee joints were harvested for histological analysis according to each experimental design.

### Collection of human tissue samples

Human OA cartilage specimens were obtained from eighteen OA patients undergoing total knee arthroplasty at the Third Affiliated Hospital of Southern Medical University (Guangzhou, China) for a diagnosis of end-stage OA, and were authorized by patients under the ethical approval of the hospital ethics committee. All patients provided complete written informed consent prior to total knee arthroplasty surgery. Patients’ information including patient ID, bed number, gender and age are recorded in Table S4. As drug treatment status is considered a key exclusion criterion for tissue explant culture study, we strictly used cartilage samples from OA patients who had no history of drug treatment for OA within the three years before total knee replacement surgery, including steroids or non-steroidal anti-inflammatory drugs (NSAIDs). It should be noted that we only used samples from female patients.

### Human OA cartilage explant culture

The cartilage specimens were cut into 1 cm^3^ pieces and washed three times with PBS (pH 7.4, Boster, AR0030, Wuhan, China) containing 1% penicillin and streptomycin (Gibco/Life Technologies, Carlsbad, CA, USA, 15140122). Then the pieces of cartilage were immediately transferred into 12-well plates filled with Dulbecco’s modified Eagle medium (DMEM)/F12 (Gibco, C11330500BT) containing 10% fetal bovine serum (Gibco, 10099141 C) and 1% antibiotics. The OOCHAS histopathology grading system and Collins’s tissue grading system were used to analyze the OA cartilage degeneration morphologically. To evaluate the effect of drugs on human OA, each compound was applied to 12-well plates containing cultured cartilage explants. Plates were treated with one of the following added to each well: 10 μmol/L NP (NP-G2-044, a Fscn1 inhibitor, Cat. No. #: HY-125506, MedChemExpress, Shanghai, China) or 2 μL DMSO (Cat. No. #: D8371, Solarbio, Beijing, China) as control, and cultured for 7 days in complete medium under a 5% CO_2_ atmosphere. The medium was replaced every other day. Each chemical treatment was performed in at least three replicates with cartilage specimens from different donors.

### Intra-articular injections

Anesthetized mice were administered 10 μL adeno-associated virus 2/5 (AAV2/5) encoding mouse Fscn1 or negative control (AAV2/5-Fscn1 or AAV2/5-control, 2.5 × 10^10^ vg, created and packaged by Hanbio Biotechnology, Shanghai, China) by intra-articular injection 2 weeks before OA-inducing DMM surgery. Additionally, two supplementary AAV injections were performed 2 and 8 weeks after DMM operation. The mice were sacrificed 6 or 10 weeks after DMM surgery and the knee joints were harvested for histological analysis. For the drug treatments of OA in mice, 10 μL of drugs: 20 mmol/L LDN-193719 (Cat. No. #: HY-12274, MedChemExpress), 10 mmol/L NP, were injected once a week via intra-articular administration into the right knee of the mice, starting one week after DMM-inducing OA surgery. The drugs are dissolved in DMSO, but diluted 10-fold in saline before injection to minimize the toxicity. As described above, the mouse knee joints were collected at 6 or 10 weeks after OA surgery.

### Safranin O staining and OA scoring

De-waxed and rehydrated tissue sections were soaked in PBS for 5 min, stained with a prepared 1% fast green solution (F7258-25G, Sigma-Aldrich, St Louis, MO, USA) for 60 s, rinsed with 3% acetic acid fixative solution for 3 s, stained with 0.5% safranin O solution (S8884-25G, Sigma–Aldrich) for 30 s, and then washed with deionized water to remove unbound stain. Finally, the sections were sealed with neutral gum after dehydrating and clearing. The histological scoring in the medial tibial plateau of the OA-model mice was quantified using the International Society for Osteoarthritis Research (OARSI) grading system (score 0–6), which required three experienced researchers, who had extensive experience in evaluating human and mouse OA, to blindly score three safranin O-stained sections for each specimen.^47^ Before grouped analysis, the mean score of the medial tibial plateau was computed for each animal. For synovitis scoring, sections were used to evaluate synovial changes using Krenn’s synovitis scoring system, where morphological parameters of synovitis including enlargement of the synovial lining layer, degree of inflammatory infiltration and activation of resident cells were graded separately (score, 0 to 3).^47,48^ Then individual scores were summed (score, 0 to 9) and classified as follows: no synovitis (score 0–1); slight synovitis (score 2–3); moderate synovitis (score 4–6) and strong synovitis (score 7–9).

### Cell culture

Mouse primary articular chondrocytes, which were isolated from femoral condyles and tibial plateaus of 3-day-old C57BL/6 J mice, were cultured in DMEM/F12 medium supplemented with 10% FBS and 1% penicillin and streptomycin in a humidified 37 °C and 5% CO_2_ atmosphere. When the cells reached 90% confluence, the P0 primary chondrocytes were digested with 0.25% Trypsin-EDTA (Gibco, 25200-072) and seeded into new dishes. P0 or P1 chondrocytes were stimulated with IL-1β (AF-211-11B, Pepro Tech, Rocky Hill, NJ, USA), strain loading or drug treatments. SW1353 cells (human chondrogenic cell line; ATCC, Manassas, VA, USA) were maintained in DMEM with 4.5 g glucose (Gibco, C11995) supplemented with 6% FBS and 1% antibiotics at 37 °C in a humidified atmosphere with 5% CO_2_. Primary chondrocytes were stimulated with 10 ng/mL IL-1β to establish a cell model of OA in vitro. Recent studies have shown that excessive mechanical stress leads to chondrocyte degeneration which also mimics an OA model in vitro.^32^ Consequently, we treated mouse primary chondrocytes with 0.5 Hz and 20% cyclic tensile strain loading via a Flexcell® FX-5000™ Tension System (Flexcell International Corp., Burlington, NC, USA) for 24 h. Cells were transfected with sh-FSCN1 (Hanbio Biotechnology), sh-DCN (Hanbio Biotechnology) or FSCN1-encoding plasmid (Hanbio Biotechnology) using Lipofectamine 3000 (Invitrogen, Carlsbad, CA, USA) following the instruction. For primary culture of human articular chondrocytes, the cells were isolated from femoral condyles and tibial plateaus of OA donors. Human articular chondrocytes were maintained in DMEM/F-12 supplemented with 10% FBS, 1% antimycotics, and 1% antibiotics, at 37 °C in a humidified atmosphere with 5% CO_2_. Cells were treated with either: 10 μmol/L NP or 2 μL DMSO for 24 h under stimulation with 10 ng/mL IL-1β.

### Actin-bundling assay

The in vitro F-actin bundling assays were performed according to the protocol provided with the Actin Binding Protein Biochem Kit™ (BK001, Cytoskeleton, Inc., Denver, CO, USA) containing G- or F-actin plus positive (α-actinin) and negative (bovine serum albumin; BSA) binding control proteins, which provided a way to obtain an answer concerning binding affinity for monomer (G-) or polymer (F-) actin. Briefly, a spin-down assay was used to measure F-actin binding, and centrifugation was performed to separate F-actin from G-actin by differential sedimentation where F-actin binding proteins co-sedimented with actin filaments and formed a pellet at the bottom of the tube. However, F-actin severing proteins, G-actin binding proteins, or non-actin binding proteins stay in the supernatant. The protein samples including pellets and supernatants were dissolved in an equivalent volume of 2× Laemmli reducing-sample buffer (Bio-Rad, 161-0737, Hercules, CA, USA). Finally, 20 μL of each sample was loaded onto a 10% sodium dodecyl sulfate-polyacrylamide gel electrophoresis (SDS-PAGE) gel and subjected to electrophoresis until the dye front reached the bottom of the gel for immunoblotting.

### Histology and immunohistochemistry

Histology is the gold standard for assessing OA in mice. Mouse knee joints were fixed with 4% paraformaldehyde (PFA, BL539A, Biosharp, Hefei, China) at 4°C for 24 h after removing skin and excess muscle, decalcified in 20% EDTA (pH 7.3) solution at room temperature (RT) for 2 weeks, then dehydrated in an automatic dehydrator (ASP300S, Leica Microsystems Ltd., Wetzlar, Germany) before embedding in paraffin. The joint specimens were cut into 4 μm thick sagittal slices and two sections were placed on every slide. About 10 tissue slides were harvested at approximately 80 μm intervals for histological and immunohistochemical staining according to standard protocols. The cultured human cartilage explants treated with the drugs mentioned above were harvested and fixed in 4% paraformaldehyde for 2 days, then 5 μm thick paraffin sections were cut and analyzed by immunohistochemical staining, as well as Safranin O & Fast Green staining. Mouse whole-mount skeleton staining of E18.5 embryos was performed with Alcian blue (A3157, Sigma–Aldrich) and Alizarin Red (A5533, Sigma-Aldrich). For immunohistochemistry (IHC), the following primary antibodies were used: FSCN1 (Abcam, ab126772, 1:200 dilution, Cambridge, UK), MMP3 (Abcam, ab52915, 1:50 dilution), and MMP13 (Abcam, ab39012, 1:100 dilution). For the quantification analysis of IHC-positivity in a standardized region of interest (ROI), the ratio of IHC-positive to total cell numbers was calculated with the number of IHC-positive cells and total number of cells counted in several ROI fields of articular cartilage for each mouse or human OA specimen in each group. Quantitation analysis of the density signal of IHC staining was performed by ImageJ (NIH, Bethesda, MD, USA) using the same signal threshold for all comparable IHC images in order to obtain non-biased results. A density value of 1 was given to the control group and all analyses were performed in at least three sections per animal from at least three animals.

### Immunofluorescence

After routine dewaxing and hydration, paraffin sections were soaked in a TE 9.0 antigen retrieval solution in a 60 °C water bath for 3 h before blocking with ready-to-use goat serum (AR0009, Boster, Wuhan, China) for 1 h at RT. Primary mouse chondrocytes cells were fixed in 4% PFA at RT for 10 min, rinsed three times in PBS to wash off the PFA, and then blocked as before. Tissue sections or cell samples were incubated at 4 °C for 16 h with the primary antibodies indicated in Table S6. The cells and slides were incubated with secondary antibodies conjugated with Alexa Fluor 488 or Alexa Fluor 594 (Invitrogen, 1:400) for 1 h at RT in the dark after carefully washing in PBS three times. F-actin was stained using Phalloidin Conjugates (Sigma-Aldrich, P5282, 100 µg/mL) for 15 min at RT in the dark. Finally, the nuclei were stained with DAPI (Sigma-Aldrich, F6057), and sections and cell samples were observed and captured at the corresponding excitation wavelength using a laser scanning confocal microscope (FV1000, Olympus, Tokyo, Japan). The immunofluorescence for G-actin staining was performed according to the manufacturer’s protocol of the Cell G-Actin Fluo Staining Kit (GMS102772, Genmed, Shanghai, China).

### Immunoprecipitation

Immunoprecipitation (IP) was performed on the protein lysates isolated from cultured chondrocytes. Aliquots containing 2–5 × 10^7^ cells were carefully washed three times with PBS (pH 7.4) and lysed in 1 mL ice-cold lysis buffer (40 mmol/L HEPES (pH 7.4), 2 mmol/L EDTA, 10 mmol/L pyrophosphate, 10 mmol/L glycerophosphate, 0.3% CHAPS and EDTA-free protease inhibitors (04693159001, Roche, Basel, Switzerland)) for 10 min at 4°C. Then, the lysates were centrifuged at a rcf of 10 000 × *g* at 4°C for 10 min and the supernatant was transferred to new centrifuge tubes. The supernatant samples were incubated with 5 μL IgG antibody (mouse anti-rabbit IgG LCS, IPKine™, A25022, Wuhan, China) and 20 μL pre-washed Protein G Sepharose™ 4 Fast Flow beads (17-0618-01, GE Healthcare, Little Chalfont, UK) under rotary agitation at 4°C for 30 min. Immediately after centrifuging samples at a rcf of 1 000 × *g* for 5 min, approximately 1 mL of the supernatant was pipetted into new 1.5 mL tubes. Then 5 μL primary antibody (Decorin or FSCN1, see Table S6) were added into the supernatant samples under rotary agitation at 4°C for 1 h and another 30 μL pre-washed Protein G Sepharose™ 4 Fast Flow beads were added as before and incubated under rotary agitation at 4°C for 4 h. After centrifugation at 1 000 × *g* for 5 min, the supernatant was discarded, and the pellet was washed 4 times with 1 mL of ice-cold PBS every time. After the last wash, the pellet was resuspended with 40 μL of 2 × SDS loading buffer, and denatured by heating on a metal plate at 100 °C for 3 min, and finally the samples were centrifuged at 10 000 × *g* for 5 min before SDS-PAGE electrophoresis.

### Immunoblotting

Primary mouse chondrocytes were subjected to five consecutive passages, IL-1β stimulation, mechanical stress, or shFscn1 lentivirus transfection. Mouse joint tissue at 6 or 10 weeks after surgical induction of OA was lysed to isolate tissue proteins by homogenizing under liquid nitrogen. Chondrocytes were treated with IL-1β, drugs, shFscn1 lentivirus, or shDecorin-lentivirus transfection. In addition, primary chondrocytes from *Fscn1*^-/-^ and *Fscn1*^*flox*/*flox*^ mice were lysed to extract protein for immunoblotting analysis. Total proteins were extracted from tissues or cultured cells using 2 × SDS lysis buffer (1 mol/L Tris-HCl (pH 6.8), 10% SDS, glycerol, bromophenol blue and protease inhibitors) on ice for 10 min and analyzed by vertical electrophoresis. Then the separated proteins were transferred to 0.2 μm PVDF membranes (ISEQ00010, Merck-Millipore, Darmstadt, Germany). Next, the membranes were blocked with 5% non-fat dried milk dissolved in 1 × tris-buffered saline (TBS) containing 0.05% Tween-20 (TBST) for 1 h at RT on a horizontal shaker and incubated at 4 °C overnight with primary antibodies (see Table S6). After removing unbound primary antibodies by washing three times with 1 × TBST, the PVDF membranes were incubated with secondary antibodies (see Table S6). Immunoreactive protein bands were detected by chemiluminescence. All immunoblotting experiments were repeated in at least three independent tests.

### RNA extraction and quantitative real-time PCR

Total RNA was extracted from chondrocytes treated with IL-1β and one of the five drugs, human chondrocytes treated with one of the five drugs, performed using Trizol Reagent (9109, TaKaRa Bio Inc., Osaka, Japan) according to the manufacturer’s protocols. cDNA was synthesized from total mRNA using a cDNA Synthesis kit (R333-01, Vazyme, Nanjing, China). The PCR primers for genes of interest are listed in Table S5 and real-time PCR or RT-PCR was performed with the LC96 system (Roche, Basel, Switzerland), using ChamQ SYBR qPCR Master Mix (Q311-02, Vazyme) according to the manufacturer’s instructions. All reactions were run in triplicate, and the 2^−ΔΔCt^ method was used for analysis of the expression of the respective genes.

### RNA sequencing

Chondrocytes cells were treated with DMSO, 10 ng/mL IL-1β, 10 ng/mL IL-1β plus 10 μmol/L NP for 24 h. Three biological replicates were used to perform RNA sequencing for each experimental group. The extraction of total RNA, sequencing operation and quantification of the final libraries were performed by BGI Sequence Company (BGI Genomics Co., Ltd, Shenzhen, China). Hierarchical cluster analysis of differentially-expressed genes (DEGs), KEGG pathway enrichment analysis of DEGs, GSEA, and volcano plots were performed to find the expression changes of genes in the different groups and samples. The RNA-seq data in this study was deposited at Gene Expression Omnibus (GEO) (http://www.ncbi.nlm.nih.gov/geo/) under accession ID GSE232611.

### ELISA for TGF-β1

Primary chondrocytes were plated in 6-well plates at an approximate confluency of 60%. Then the cells were treated with 10 ng/mL interleukin-1 beta (IL-1β). In parallel, the expression of FSCN1 was modulated by knockdown using shFSCN1 and by overexpression through plasmid-mediated transfection. Sixty hours post-treatment, the culture supernatants were harvested for the quantification of mouse TGF-β1 using an enzyme-linked immunosorbent assay (ELISA) kit for TGF-β1 (MEIMIAN, Cat#MM-0135M2, Jiangsu) according to the manufacturer’s instructions.

### Chondrocyte Pellet culture

Primary chondrocytes were seeded in 6-well plates at an approximate confluency of 60%. The cells were subsequently treated with 10 ng/mL interleukin-1 beta (IL-1β). Concurrently, the expression of FSCN1 was modulated through knockdown using shFSCN1, and overexpression facilitated by plasmid-mediated transfection. Sixty hours post-treatment, 4 × 10^5^ cells from each experimental group were centrifuged for 5 min at 150 × *g* in 1.5 mL polystyrene tubes. The culture medium was replenished every other day. After a period of 10 days, the resulting pellets were carefully collected from the tubes and fixed with 4% formaldehyde for 12 h at 4 °C, followed by dehydration in a graded sucrose series: 10% for 12 h, 20% for an additional 12 h, and finally, 30% for a further 12 h (totaling 48 h). The tissue samples were then embedded in OCT compound (Leica, Cat#3801480, Germany) and frozen sections of 5 μm thickness were prepared using a cryostat (Leica, Cat#CM3050S, Germany) according to a previously described protocol (PMID: 36696903). The sections were subsequently stained with Alcian blue or subjected to immunofluorescence analysis, and examined using a BX43 microscope (Olympus, Japan) or an Olympus FV1200 confocal microscope.

### Immunoprecipitation-LC-MS/MS analysis

Mouse primary chondrocytes (1 ×10^7^) were lysed in a buffer containing 25 mmol/L Tris, 150 mmol/L NaCl, 1 mmol/L EDTA, 1% NP-40, 5% glycerol, 1 mmol/L NaF, 1 mmol/L PMSF, and protease inhibitors. Clarified lysates (1 mg protein) underwent immunoprecipitation with anti-DCN antibody or control IgG antibodies coupled to Protein A/G Plus Agarose beads. The precipitated proteins were separated by SDS-PAGE, visualized by Coomassie staining, and subjected to in-gel trypsin digestion followed by liquid chromatography-tandem mass spectrometry analysis on a Q Exactive mass spectrometer coupled to UPLC, which was performed at the Fitgene Biotechnology Company (Guangzhou, China). Peptides were loaded onto a reversed-phase column (75 μm x 150 mm, C18) at 300 μL/min and ionized by nanospray. MS/MS was performed at 70 000 resolutions for intact peptides and 17 500 for fragments. Data-dependent acquisition involved one MS scan followed by 20 MS/MS scans of the top 20 precursors ( > 1E4 ions, 30 s exclusion). The AGC target captured ions up to 1E5 intensity within m/z 350–1 800. Protein identification used MASCOT to search the UniProt human database, allowing one missed cleavage, with carbamidomethylation as fixed and oxidation as variable modifications (20 × 10^6^ peptide, 0.6 Da fragment tolerance). Monoisotopic mass calculation was used at *P* < 0.05 significance. Proteins from the IgG precipitate were considered contaminants and excluded.

### RPLC-MS based DIA proteomics analysis

Proteomics analysis was performed on human osteoarthritic (OA) and control cartilage isolated from patients who underwent knee replacement surgery, as well as protein samples extracted from mouse chondrocytes with or without FSCN1 knockdown by shRNA and treated with IL-1β (*n* = 3 independent biological replicates per group). Samples were stored at −80 °C until shipment to the Fitgene Biotechnology Company (Guangzhou, China) for Reverse-Phase Liquid Chromatography-Mass Spectrometry based Data-Independent Acquisition (RPLC-MS DIA) proteomics analysis.

First, protein samples underwent quantification. A standard curve was prepared using 0, 1, 2, 4, 8, 12, 16, and 20 μg of BSA, with 2 μL of sample in duplicate. Following the instructions of the Beyotime BSA assay kit, samples were vortexed for 20 s to ensure thorough mixing, heated at 60 °C for 30 min, and absorbance was measured to construct the standard curve.

Next, protein samples were subjected to FASP digestion. After quantification, 30 μg of protein solution was transferred to a centrifuge tube, and 4 μL of TCEP Reducing Reagent was added, followed by incubation at 60 °C for 1 h. Subsequently, 2 μL of MMTS Cysteine-Blocking Reagent was added, and the mixture was incubated at room temperature for 30 min. The reduced and alkylated protein solution was then transferred to a 10 K ultrafiltration unit and centrifuged at 12 000 × *g*, 4 °C for 20 min. The flow-through in the collection tube was discarded. 8 mol/L urea (pH 8.5) (100 μL) was added, and the centrifugation step was repeated twice, discarding the flow-through each time. Next, 0.25 mol/L TEAB (pH 8.5) (100 μL) was added, and the centrifugation step was repeated three times, discarding the flow-through each time. A new collection tube was used, and 50 μL of 0.5 mol/L TEAB was added to the ultrafiltration unit, followed by the addition of trypsin (trypsin: protein ratio of 1:50). The mixture was incubated at 37 °C overnight. The next day, additional trypsin (trypsin: protein ratio of 1:100) was added, and the mixture was incubated at 37 °C for 4 h. After centrifugation at 12 000 × *g* for 20 min, the digested peptide solution was collected in the bottom of the collection tube. Subsequently, 50 μL of 0.5 mol/L TEAB was added to the ultrafiltration unit, and the centrifugation step was repeated at 12 000 × *g*, 4 °C for 20 min. The flow-through was combined with the previous collection, resulting in a total of 100 μL of the digested sample.

Next, the digested sample underwent proteomic analysis using second-dimension reverse-phase liquid chromatography-mass spectrometry (RPLC-MS). The mobile phase information was as follows: Mobile phase A: 0.1% formic acid; Mobile phase B: 0.1% formic acid, 80% acetonitrile. A home-made tip-column (150 μm × 250 mm, 3 μm-C18) was used for chromatographic separation. Peptides were dissolved in the sample solvent (0.1% formic acid), and an appropriate amount of iRT reagent was added. After thorough vortexing, samples were centrifuged at 13 500 r/min, 4 °C for 20 min, and the supernatant was transferred to an autosampler vial. 3 μg of sample was injected for high-performance liquid chromatography separation at a flow rate of 600 nL/min. The digested products, separated by high-performance liquid chromatography, were analyzed by an Orbitrap Fusion Lumos mass spectrometer (Thermo Scientific) in positive ion mode for 120 min. The specific mass spectrometry parameters were as follows: Primary MS parameters: Resolution: 120 000; AGC target: 4e6; Maximum IT: 50 ms; Scan range: 350 to 1 250 m/z. DIA MS parameters: Resolution: 30 000; AGC target: 5e6; Maximum IT: 50 ms; NCE/stepped NCE: 31.

The fold change (FC) was computed as the ratio of the mean relative quantitative values of proteins between two sample groups. A T-test was performed on these values to assess the statistical significance of differences, yielding a *P*-value that acted as an index of significance. The significance threshold was predefined at a *P*-value of less than 0.05. To satisfy the normal distribution prerequisite for the T-test, the relative quantitative protein values were log_2_-transformed prior to analysis. From this differential analysis, a *P*-value less than 0.05 indicated a significant change: a differential expression level increase greater than 1.5-fold was considered significant upregulation, and a decrease below 1/1.5-fold was considered significant downregulation.

### Statistical analysis

Statistical analysis was performed using SPSS version 20.0 software, and graphs were generated using GraphPad Prism 8.0. Data normality was assessed using the Kolmogorov-Smirnov and Shapiro-Wilk tests. For comparisons between two independent groups, the unpaired, two-tailed Student’s t-test or Mann-Whitney rank-sum test was used, depending on data normality. One-way ANOVA with Tukey’s post hoc test or the Kruskal-Wallis test with Dunn’s multiple comparisons test was used for multiple comparisons, as appropriate. All statistical tests were two-sided, and a *P*-value < 0.05 was considered statistically significant. The sample size for each experiment was indicated in the figure legend and was determined to ensure adequate statistical power based on the observed effect sizes and available samples. All experiments were performed in triplicate, with at least three independent biological replicates, including cell, histology, and immunohistochemistry experiments.

## Supplementary information

Supplementary Materials -2024-07-30

Uncropped gel-1

Uncropped gel-2

Supplementary table 1

Supplementary table 2

Supplementary table 3

## Data Availability

The sequence data reported in this paper have been deposited in the Gene Expression Omnibus (GEO) database with an accession no. GSE232611. All other data are included in the article and Supplementary Materials.
