# Supplementary material for: Targeting Fascin1 maintains chondrocytes phenotype and attenuates osteoarthritis development
Source: Bone Res. 2024 Sep 4;12:50. doi: 10.1038/s41413-024-00357-1 (PMC11374990; doi:10.1038/s41413-024-00357-1)
Supplement: Supplementary file 1 — Supplementary Materials -2024-07-30 [file 41413_2024_357_MOESM1_ESM.docx]

**Targeting Fascin1 maintains chondrocytes phenotype and attenuates osteoarthritis development**

Panpan Yang1, †, Yun Xiao1, †, Liangyu Chen1, †, Chengliang Yang2, Qinwei Cheng1, Honghao Li1, Dalin Chen1, Junfeng Wu1, Zhengquan Liao1, Changsheng Yang1, Chong Wang3, Hong Wang1, Bin Huang1, Ee Ke4, Xiaochun Bai1, 5, *, Kai Li1, *

Corresponding to Kai Li or Xiaochun Bai, E-mail: lk516433415@smu.edu.cn; baixc15@smu.edu.cn.

**Supplementary Figures**

**
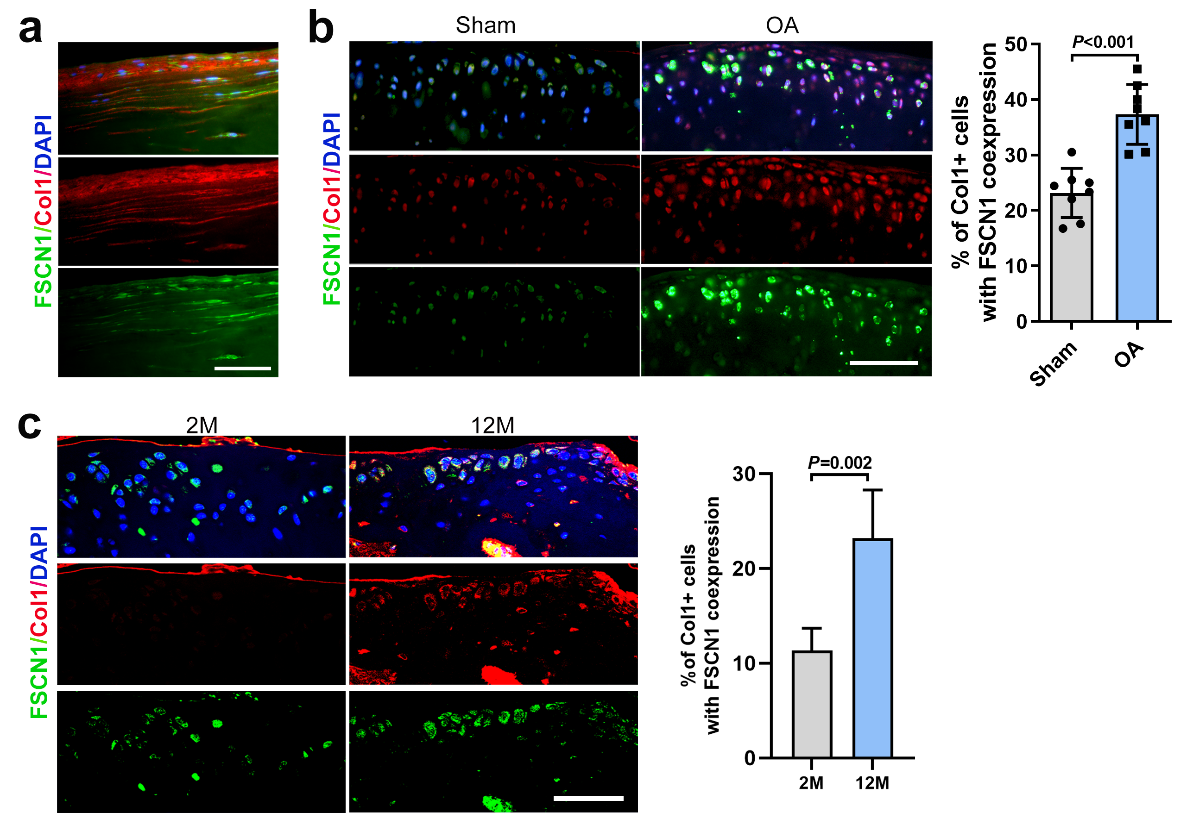
**

**Supplementary Figure 1.** **FSCN1 positive cells locate in the superficial area of cartilages from human OA, DMM-induced OA and aged mice.** a. IF staining of FSCN1 (green) and Collagen type I (red) in cartilage from OA patients. Scale bars, 50μm. b-c. IF staining and quantification of FSCN1 (green) and Collagen type I (red) in articular cartilage from mice with sham or DMM-induced OA at 6 weeks post-surgery (b), and from young and aged mice (c). n = 5 mice per group. All data are presented as means ± SEM. Unpaired Student’s t test was performed.

**
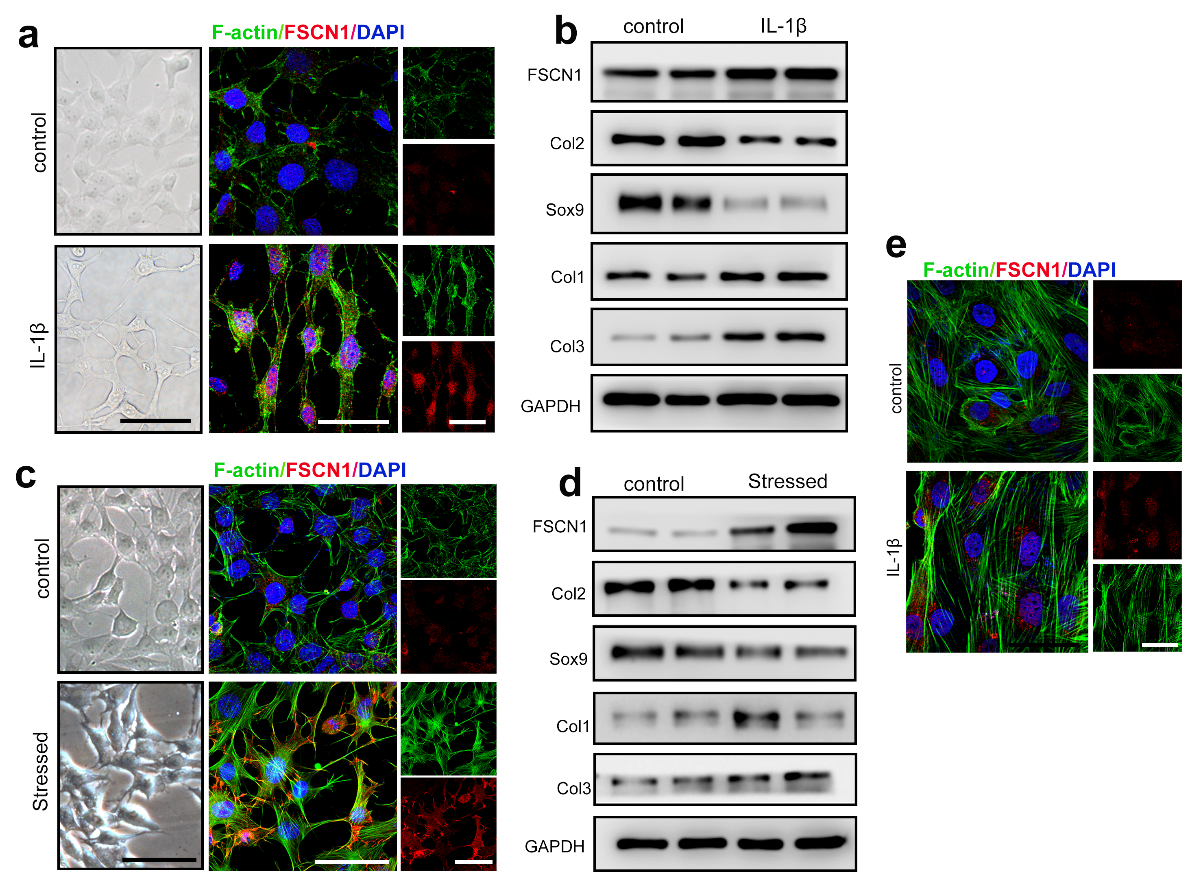
**

**Supplementary Figure 2.** **Upregulated FSCN1 is associated with chondrocyte dedifferentiation.** a-b. Optical microscopy, IF staining of FSCN1 (red) and phalloidin (green) staining for F-actin structures (a), Western blot analysis of protein levels of FSCN1, Collagen II, Sox9, Collagen I and III (b) in mouse primary chondrocytes treated with saline or IL-1β (10ng/ml) for 24hours. n = 3. c-d. Optical microscopy, IF staining of FSCN1 (red) and phalloidin (green) staining for F-actin structures (c), Western blot analysis of protein levels of FSCN1, Collagen II, Sox9, Collagen I and III (d) in mouse primary chondrocytes treated with elongation strain loading (10%) for 24 hours. Scale bar, 50μm. n = 3 independent experiments. e. IF staining of FSCN1 (red) and phalloidin (green) staining for F-actin structures in the human chondrocyte cell line SW1353 treated with saline or IL-1β (10ng/ml) for 24 hours. Scale bar, 50μm.

**
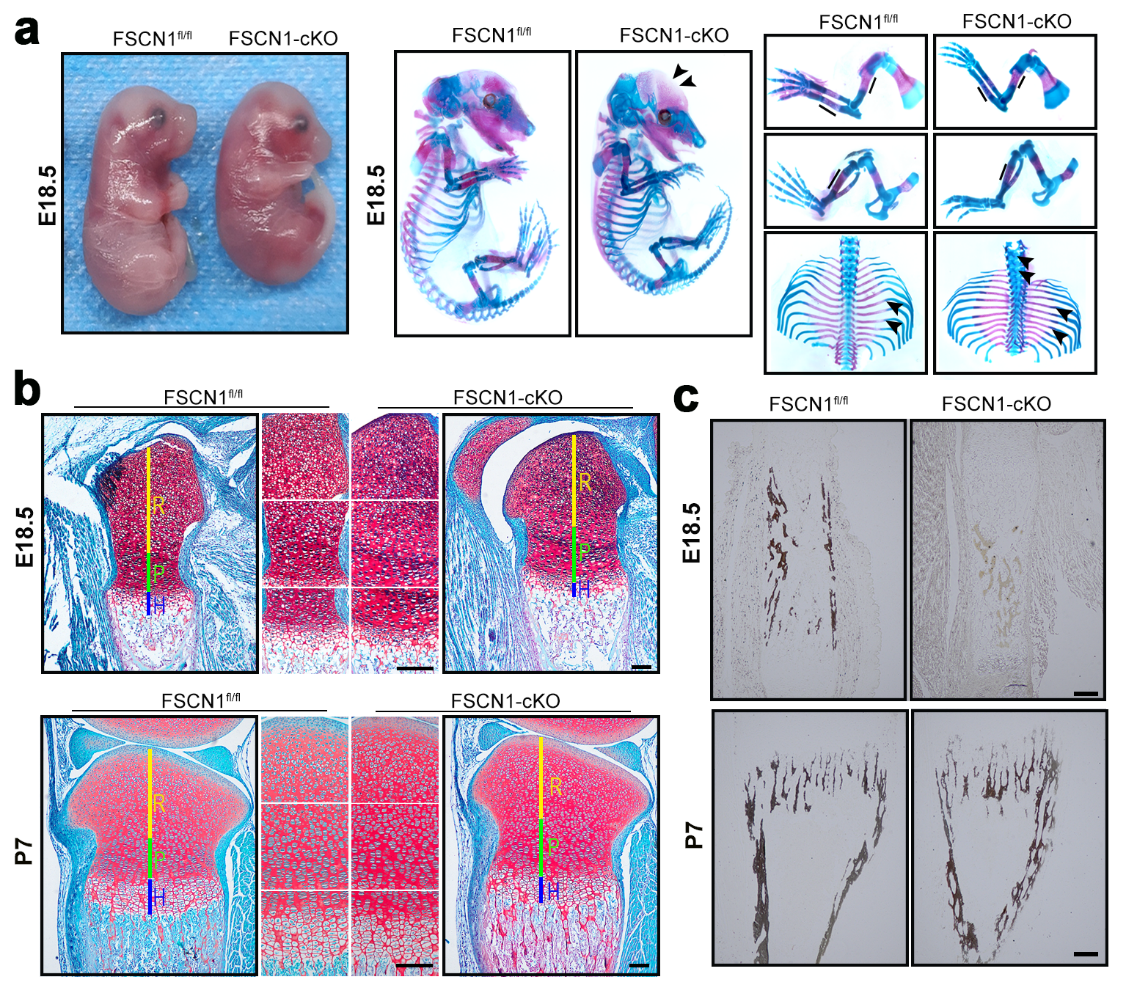
**

**Supplementary Figure 3.** **Chondrocyte-specific FSCN1 knockout causes skeletal development defects in mice.** a. Gross appearance images, Alcian blue and alizarin red staining of skeletons of control mice and FSCN1-cKO mice at embryonic day 18.5 (E18.5); n = 5. b-c. SOFG and Von kossa staining of sagittal sections of tibiae from control mice or FSCN1-cKO mice at E18.5 and P7. R, resting zone, P, proliferation zone, H, hypertrophic zone; n = 3. Scale bars, 50μm.


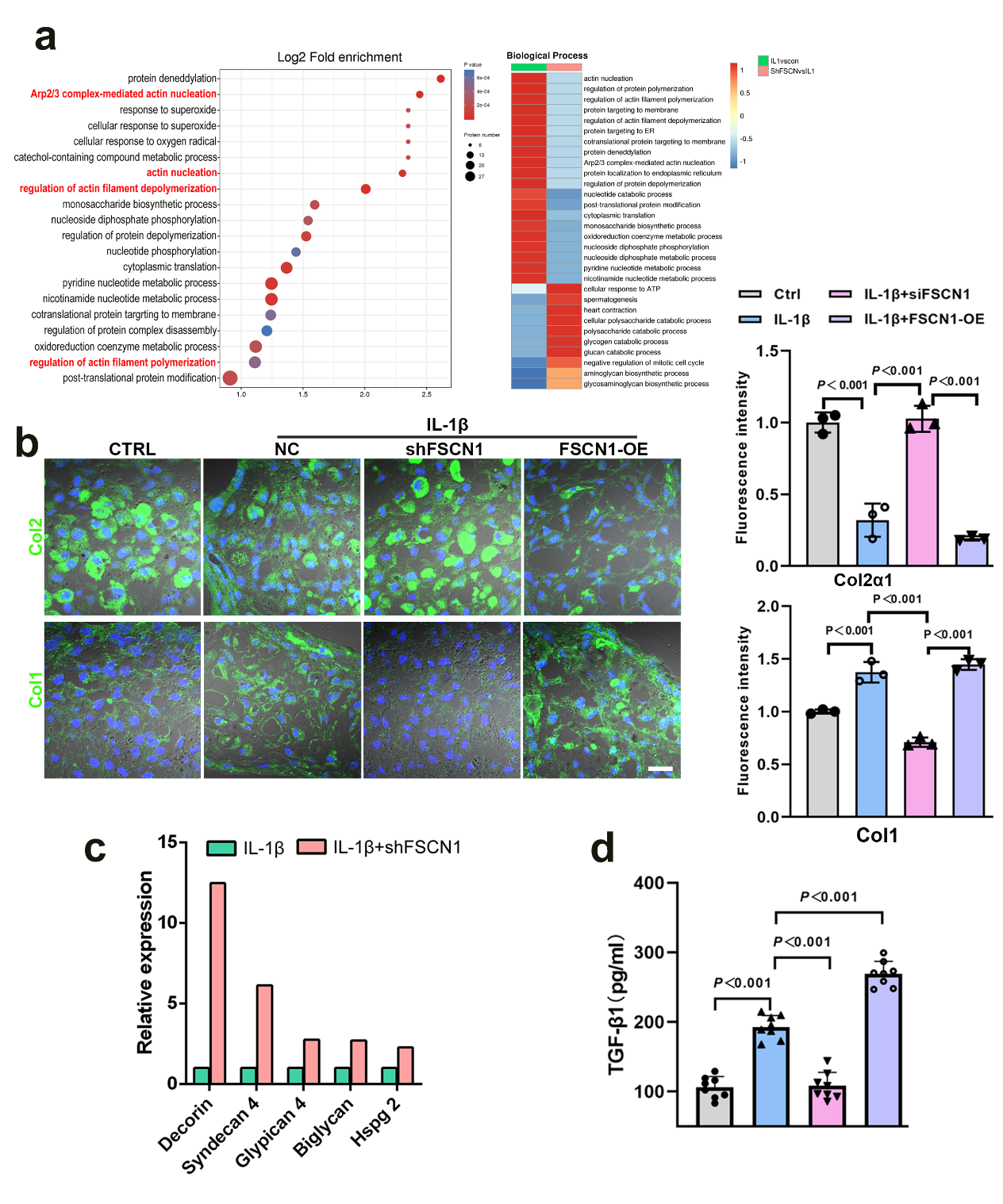


**Supplementary Figure 4.** **FSCN1 promotes actin polymerization and interacted with DCN in chondrocytes.** a. Functional enrichment analysis showing all changing biological processes in chondrocytes with IL-1β plus FSCN1 knockdown versus IL-1β treatment alone. b. IF staining and quantification of Col2 and Col1 in high-density pellet cultured chondrocytes with control, IL-1β (10ng/ml), IL-1β plus FSCN1 knockdown or IL-1β plus FSCN1 overexpression treatment for 10 days. c. Quantitative analysis of upregulated proteoglycans in the proteome-wide screen for differentially-expressed proteins (>1.5-fold) by analyzing chondrocytes treated with IL-1β or IL-1β plus FSCN1 knockdown treatment. d. Elisa analysis of TGFβ1 in medium supernatant from cultured chondrocytes with control, IL-1β (10ng/ml), IL-1β plus FSCN1 knockdown or IL-1β plus FSCN1 overexpression treatment for 24 hours. Scale bars, 50μm. All data are presented as means ± SEM.


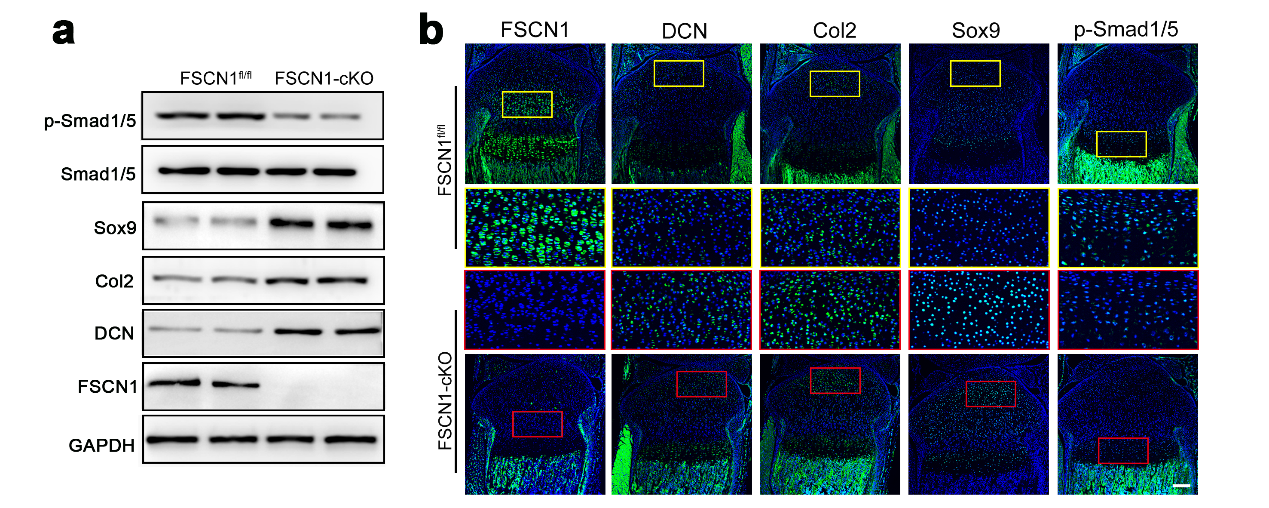


**Supplementary Figure 5.** **Chondrocyte-specific FSCN1 knockout inhibits chondrocyte differentiation and ALK1/Smad1/5 signaling *in vivo*.** a. Western blot analysis of protein levels of p-Smad1/5, Smad1/5, Sox9, Collagen type II, DCN and FSCN1 in chondrocytes from control mice and FSCN1-cKO mice; n = 3 independent experiments. b. IF staining of FSCN1, DCN, Collagen type II, SOX9 and p-Smad1/5 in sagittal sections of tibiae from control mice or FSCN1-cKO mice at P7; n = 3. Scale bars, 50μm.

**
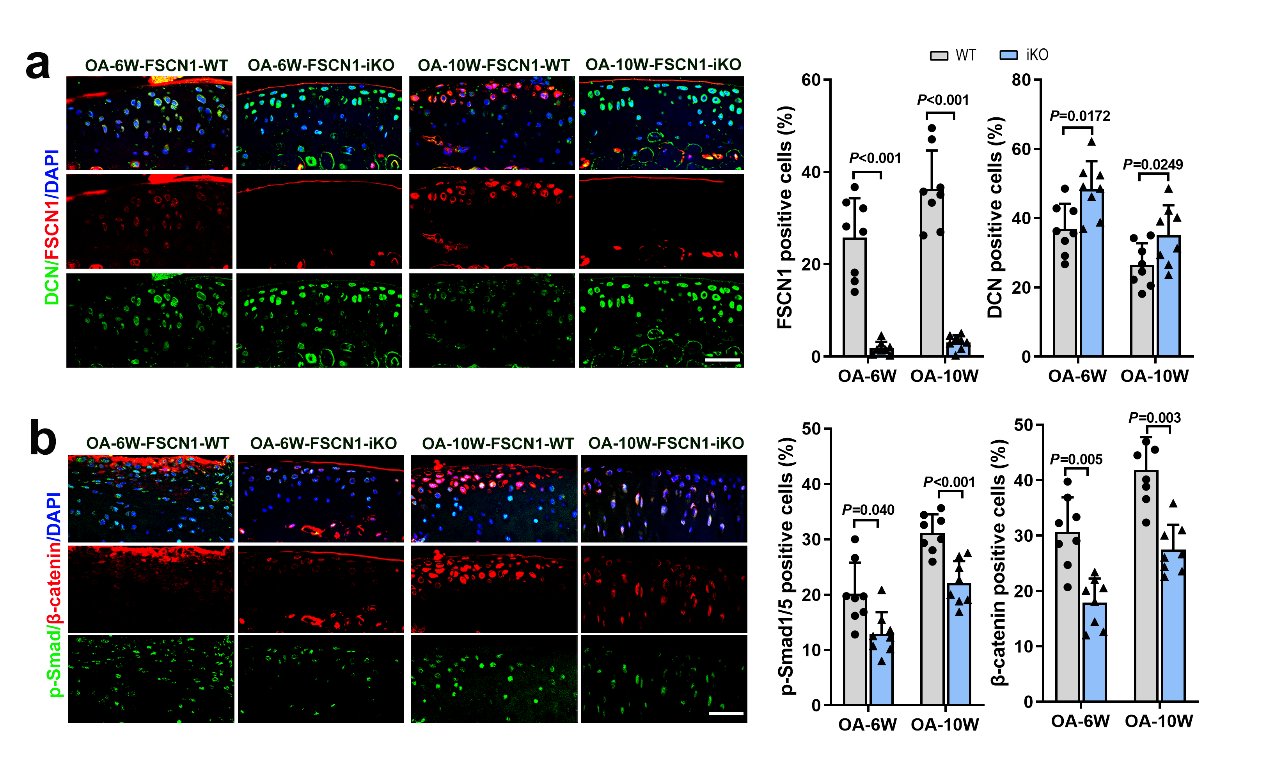
**

**Supplementary Figure 6. Targeted deletion of FSCN1 in chondrocytes prevents OA development via inhibiting ALK1/Smad1/5 signaling.** a-b. IF staining and quantification of FSCN1 (red) and DCN (green) (a), p-Smad1/5 (green) and β-catenin (red) (b) in articular cartilage from FSCN-WT and FSCN1-iKO mice at 6- or 10-weeks post-surgery. Quantified results of each set of data are shown below; n = 8. Scale bars, 50 μm. All data are presented as means ± SEM.

**
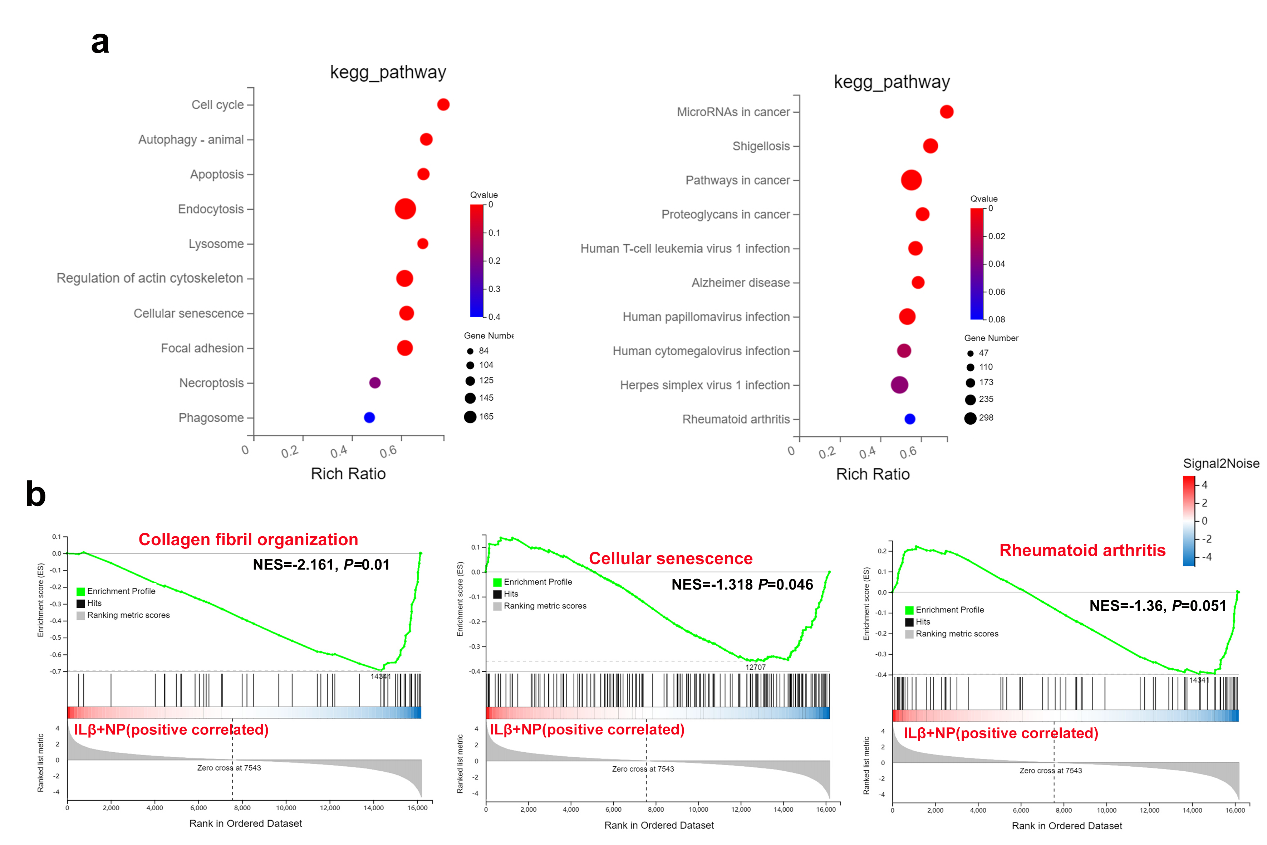
**

**Supplementary Figure 7.** **Analysis of RNA-sequencing results in chondrocytes with or without NP-G2-044 treatment.** a-b. KEGG and GSEA analysis demonstrating collagen fibril organization, cellular senescence and rheumatoid arthritis signaling pathways downregulated in chondrocytes treated IL-1β plus NP-G2-044 compared with IL-1β alone.

**
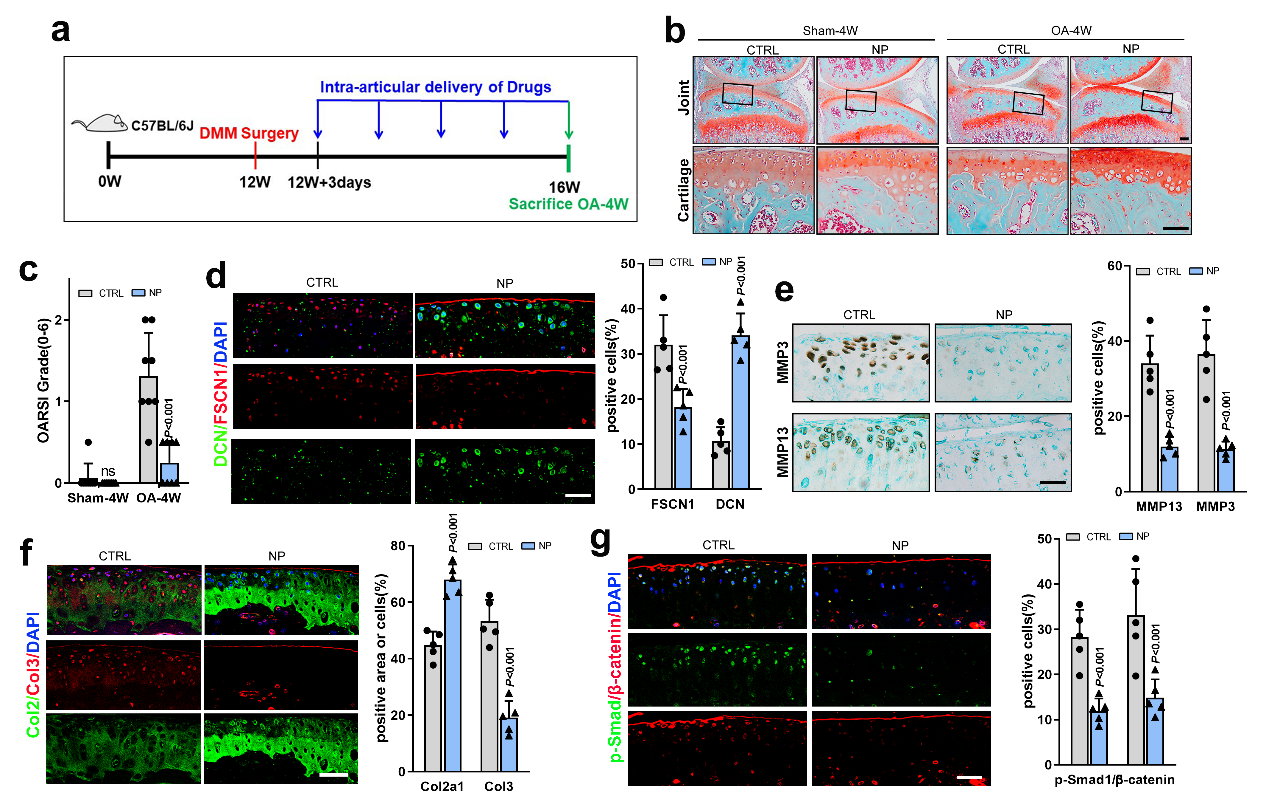
**

**Supplementary Figure 8.** **FSCN1 inhibitor ameliorates OA onset in mice with DMM-induced OA.** a. Twelve-week-old C57BL6/J mice underwent DMM or sham surgery, and saline (control) or NP (NP-G2-044, 10 mM) was injected into the knee joints every week after surgery, started at three days after surgery. The knees were harvested at 4 weeks postoperatively for histological analysis. n = 8. b. SOFG staining of joints from mice with DMM-induced OA and treated with control or NP at 4-weeks post-surgery. The insets in the images are shown as magnified images in the bottom row. c. Cartilage destruction (OARSI grades) was determined by SOFG staining and scored; n = 8. d-g. IF staining and quantification of DCN (green) and FSCN1 (red) (d), IHC staining and quantification of MMP3 and MMP13 (e), IF staining and quantification of Collagen type II (green) and III (red) (f), p-Smad1/5 (green) and β-catenin (red) (g) in articular cartilage from control mice and those treated with NP at 4-weeks post-surgery; n = 5. Scale bars, 50μm. All data are presented as means ± SEM. One-way ANOVA was performed followed by Tukey’s post-hoc analysis, with p values indicated in figure.

**
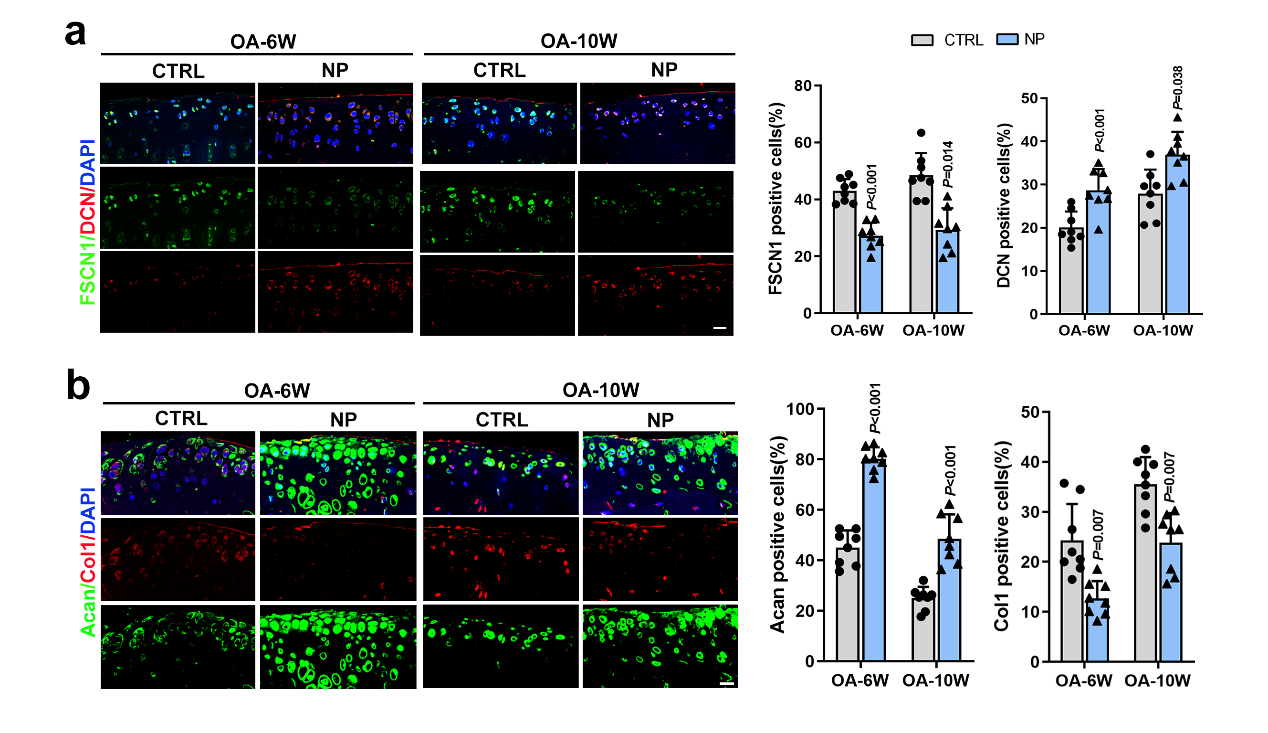
**

**Supplementary Figure 9.** **FSCN1 inhibitor ameliorates experimental OA in mice.** a. IF staining and quantification of FSCN1 (green) and DCN (red) in articular cartilage from DMM-induced OA mice treated with control or NP at 10-weeks post-surgery. b. IF staining and quantification of ACAN (green) and Col1 (red) in synovium from DMM-induced OA mice treated with control or NP at 10-weeks post-surgery; n = 8 mice per group. Scale bars, 50μm. All data are presented as means ± SEM. One-way ANOVA was performed followed by Tukey’s post-hoc analysis, with p values indicated in figure.

**Table S1 to S3 related to RNA-sequencing and proteomic analysis have been provided in supplementary excel files:**

Table S1-Differentially expressed proteins in proteomic analysis of human cartilages;

Table S2- Differentially expressed proteins in proteomic analysis;

Table S3-DEGs and KEGG pathway enrichment in RNA-Seq of IL vs IL+NP.

**Table S4** **Information of osteoarthritis (OA) patients**

| **Name** | **ID** | **Age** | **Gender** | **Time** | **Bed number** | | **Morphologically Grade** |
| --- | --- | --- | --- | --- | --- | --- | --- |
| Zheng Lan fang | P222930 | 57 | female | 2021/3/19 | 32 | Early stage | |
| Zhuo Cai qin | 1364230 | 78 | female | 2020/5/14 | 25 | End stage | |
| Zhong Xing yue | 1364933 | 75 | female | 2020/5/22 | 31 | End stage | |
| Li Li yi | 1364988 | 70 | female | 2020/5/22 | 41 | Early and End stage | |
| Wang Xiao ying | 1366121 | 60 | female | 2020/5/29 | 36 | Early and End stage | |
| Du Gui ling | 1366825 | 60 | female | 2020/6/5 | 11 | Early stage | |
| Ye Run feng | 1368542 | 75 | female | 2020/8/11 | 19 | End stage | |
| Chen Dai ying | 1369318 | 73 | female | 2020/9/7 | 14 | Early and End stage | |
| Du Xing | P222665 | 65 | female | 2021/3/22 | 14 | Early and End stage | |
| Wang Feng lan | P219803 | 68 | female | 2021/3/22 | 2 | Early and End stage | |
| Huang Ke ai | P222800 | 71 | female | 2021/3/22 | 16 | Early and End stage | |
| He Li ping | P206654 | 58 | female | 2021/3/24 | 43 | Early stage | |
| Hu Qiao | P222953 | 67 | female | 2021/3/24 | 37 | Early and End stage | |
| Lun Feng yan | P222873 | 64 | female | 2021/3/24 | 15 | Early and End stage | |
| Pan Jing xian | P222944 | 68 | female | 2021/3/24 | 18 | End stage | |
| Yu Zhen hua | P223033 | 61 | female | 2021/3/26 | 22 | Early and End stage | |
| Zhou Hua xiang | P223015 | 70 | female | 2021/3/26 | 31 | End stage | |
| Huang Li wen | P222957 | 66 | female | 2021/3/26 | 34 | Early and End stage | |

**Table S****5 List of RT-PCR primers**

| **Gene** | **Forward primer** | **Reverse primer** |
| --- | --- | --- |
| *m-Col1α1* | 5’-GCTCCTCTTAGGGGCCACT-3’ | 5’-CCACGTCTCACCATTGGGG-3’ |
| *m-Col3α1* | 5’-CTGTAACATGGAAACTGGGGAAA-3’ | 5’-CCATAGCTGAACTGAAAACCACC-3’ |
| *m-Col2α1* | 5’-GGGAATGTCCTCTGCGATGAC-3’ | 5’-GAAGGGGATCTCGGGGTTG-3’ |
| *m-GAPDH* | 5’-TGGCCTTCCGTGTTCCTAC-3’ | 5’-GAGTTGCTGTTGAAGTCGCA-3’ |
| *m-Fscn1* | 5’-GACTGCGAAGGTCGCTACC-3’ | 5’-CTGATCGGTCTCTTCATCCTGA-3’ |
| *m-DCN* | 5’-TCTTGGGCTGGACCATTTGAA-3’ | 5’-CATCGGTAGGGGCACATAGA-3’ |
| *m-Sox9* | 5’-TCTTGGGCTGGACCATTTGAA-3’ | 5’-CATCGGTAGGGGCACATAGA-3’ |
| *h-Col1α1* | 5’-GAGGGCCAAGACGAAGACATC-3’ | 5’-CAGATCACGTCATCGCACAAC-3’ |
| *h-Col2α1* | 5’-CCAGATGACCTTCCTACGCC-3’ | 5’-TTCAGGGCAGTGTACGTGAAC-3’ |
| *h-DCN* | 5’-ATGAAGGCCACTATCATCCTCC-3’ | 5’-GTCGCGGTCATCAGGAACTT-3’ |
| *h-Sox9* | 5’-AGCGAACGCACATCAAGAC-3’ | 5’-CTGTAGGCGATCTGTTGGGG-3’ |
| *h-GAPDH* | 5’-GGAGCGAGATCCCTCCAAAAT-3’ | 5’-GGCTGTTGTCATACTTCTCATGG-3’ |
| *h-Col3α1* | 5’-TTGAAGGAGGATGTTCCCATCT-3’ | 5’-ACAGACACATATTTGGCATGGTT-3’ |
| *h-Fscn1* | 5’-CCAGGGTATGGACCTGTCTG-3’ | 5’-GTGTGGGTACGGAAGGCAC-3’ |

**Table S6 List of Antibodies**

| **Antibody** | **Brand** | **Species** | **Cat. No.** | **Dilution** |
| --- | --- | --- | --- | --- |
| Fascin1 | Abcam | Rabbit | ab126772 | 1:200 for IF/ICC |
| Fascin1 | CST | Mouse | 99978 | 1:100 for IHC |
| Fascin1 | CST | Mouse | 54545 | 1:1000 for WB |
| MMP3 | Abcam | Rabbit | ab52915 | 1:50 for IHC |
| MMP13 | Abcam | Rabbit | ab39012 | 1:100 for IHC |
|  |  |  |  | 1:5000 for WB |
| Collagen III | Abcam | Mouse | ab6310 | 1:100 for IF/ICC |
|  |  |  |  | 1:2000 for WB |
| β-Catenin | CST | Mouse | 37447 | 1:500 for IF/ICC |
| p-Smad1/5 | CST | Rabbit | 9516 | 1:100 for IF/ICC |
| Smad1/5 | Abcam | Rabbit | 80255 | 1:1000 for WB |
| Decorin | Abcam | Rabbit | ab175404 | 1:100 for IF/ICC |
|  |  |  |  | 1:1000 for WB |
| Decorin | Abcam | Mouse | ab268048 | 1:100 for IF/ICC |
| Collagen II | Abcam | Rabbit | ab34712 | 1:100 for IHC/IF |
| Collagen II | Abcam | Rabbit | ab188570 | 1:3000 for WB |
| ACAN | Abcam | Rabbit | ab36861 | 1:100 for IHC/IF |
| Sox9 | Abclonal | Rabbit | a19710 | 1:100 for IHC/IF |
|  |  |  |  | 1:2000 for WB |
| Col10 | Abcam | Rabbit | ab58632 | 1:200 for IHC/IF |
| Ki67 | Abcam | Rabbit | ab15580 | 1:500 for IF/ICC |
| Collagen I | CST | Mouse | 66948 | 1:100 for IF/ICC |
| GAPDH | Ray Antibody | Mouse | RM2002 | 1:3000 for WB |
| Anti-Rabbit IgG | Ray Antibody | Rabbit | RM3002 | 1:5000 for WB |
| Anti-Mouse IgG | Ray Antibody | Mouse | RM3001 | 1:5000 for WB |
| Anti-Rabbit IgG LCS | IPKine™ | Mouse | A25022 | 1:200 for IP |
| Anti-Rabbit IgG LCS | CST | Mouse | 93702 | 1:200 for IP |
| Anti-Mouse IgG LCS | IPKine™ | Goat | A25012 | 1:200 for IP |
| Anti-Rabbit Alexa Fluor™ 488 | Invitrogen | Goat | a11008 | 1:400 for IF/ICC |
| Anti-Rabbit Alexa Fluor™ 594 | Invitrogen | Goat | a11012 | 1:400 for IF/ICC |
| Anti-Mouse Alexa Fluor™ 488 | Invitrogen | Donkey | a21202 | 1:400 for IF/ICC |
| Anti-Mouse Alexa Fluor™ 594 | Invitrogen | Donkey | a21203 | 1:400 for IF/ICC |
| Phallodine-FITC | Sigma-Aldrich |  | P5282 | 1:200 for ICC |
