# Supplementary figures and images for: Targeting Fascin1 maintains chondrocytes phenotype and attenuates osteoarthritis development
Source: Bone Res. 2024 Sep 4;12:50. doi: 10.1038/s41413-024-00357-1 (PMC11374990; doi:10.1038/s41413-024-00357-1)

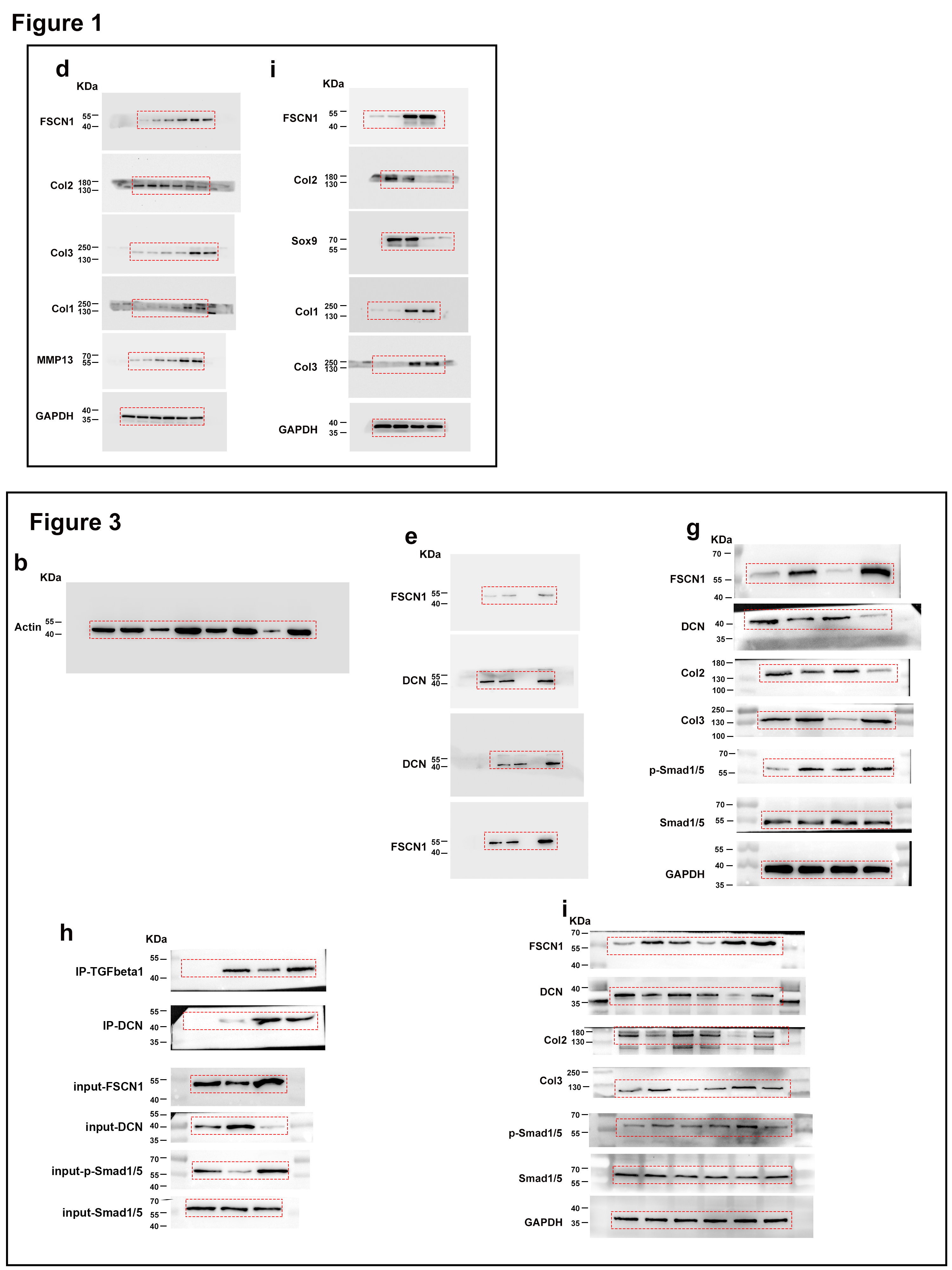

Supplement: Supplementary file 2 — Uncropped gel-1 [file 41413_2024_357_MOESM2_ESM.tif]

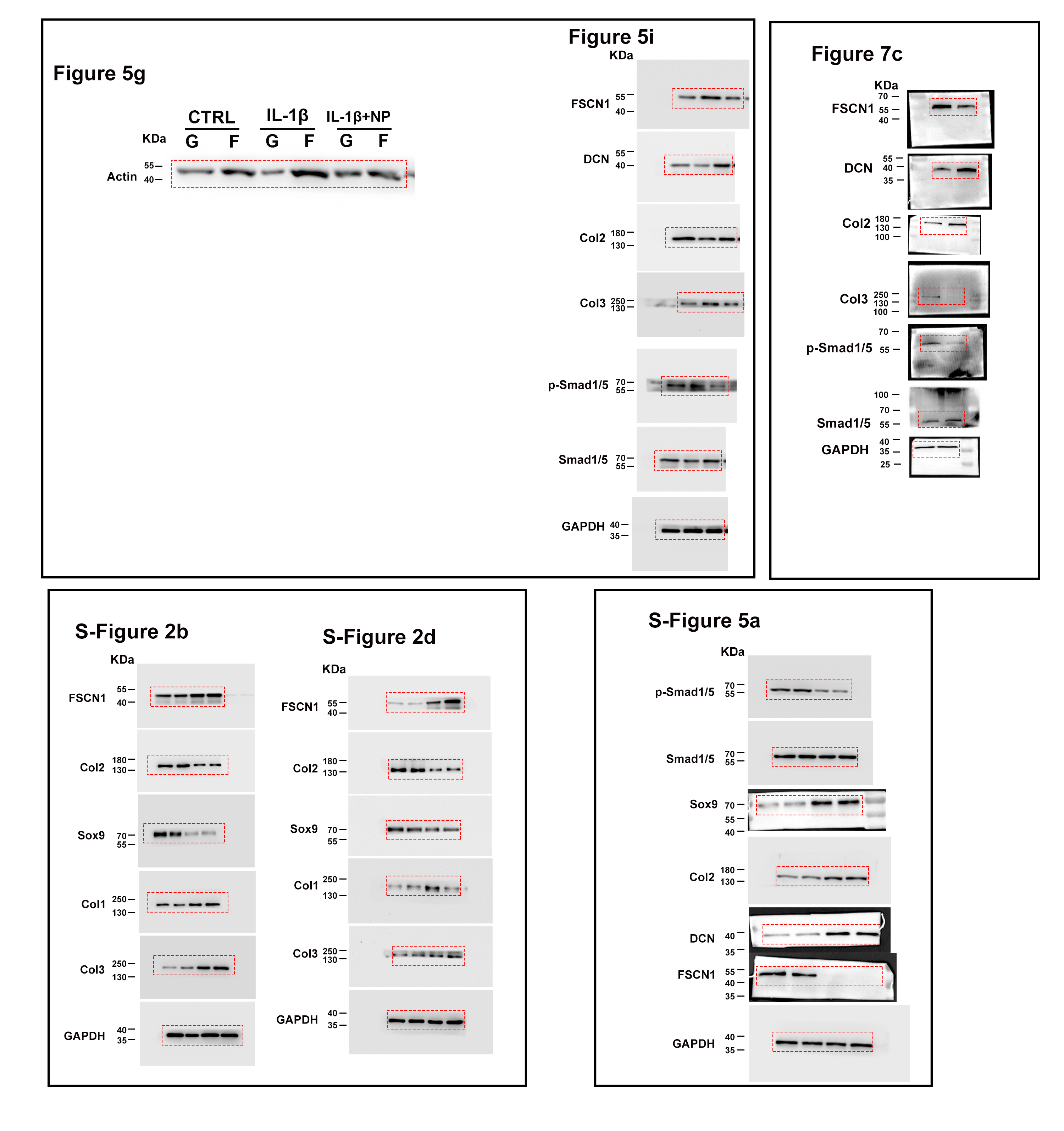

Supplement: Supplementary file 3 — Uncropped gel-2 [file 41413_2024_357_MOESM3_ESM.tif]
